# Compartment-Specific iPSC-Derived Cardiomyocytes and Organoids: Differentiation Strategies and Applications in Cardiovascular Disease Modeling

**DOI:** 10.3390/cells15161460

**Published:** 2026-08-14

**Authors:** Aaron D. Argall, Rabina Shrestha, Jiyoon Lee, Ming-Tao Zhao

**Affiliations:** 1 Center for Cardiovascular Research, Abigail Wexner Research Institute, Nationwide Children’s Hospital, Columbus, OH 43205, USA; aaron.argall2@nationwidechildrens.org (A.D.A.); rabina.shrestha@nationwidechildrens.org (R.S.); jiyoon.lee@nationwidechildrens.org (J.L.); 2 The Heart Center, Nationwide Children’s Hospital, Columbus, OH 43205, USA; 3 Biomedical Sciences Graduate Program, The Ohio State University College of Medicine, Columbus, OH 43210, USA; 4 Department of Pediatrics, The Ohio State University College of Medicine, Columbus, OH 43210, USA

**Keywords:** induced pluripotent stem cell, cardiomyocyte, congenital heart disease, cardiac development, ventricle, atria, atrioventricular canal, outflow tract

## Abstract

Compartment-specific induced pluripotent stem cell (iPSC)-derived cardiomyocytes provide a powerful resource to study cellular and molecular underpinnings of congenital heart disease (CHD) and acquired cardiovascular disease (CVD). Human heart development requires coordinated and complex regulation of key signaling pathways including Notch, BMP, Wnt, Nodal, and Shh during each step of heart morphogenesis. Compartment-specific cardiac cells for modeling the various structures in the heart can be generated by fine tuning these signaling pathways in a sequential manner thereby mimicking spatiotemporal regulation during embryonic heart morphogenesis. In this review, we provide a brief overview of key signaling pathways that are responsible for forming the distinct structures of the heart originating from the first heart field (FHF) and second heart field (SHF). We then summarize recent differentiation protocols that leverage key heart-development related signaling molecules to generate compartment-specific cardiomyocytes and organoids for disease modeling and therapeutic development in cardiovascular disease.

## 1. Introduction

**Figure 1:**
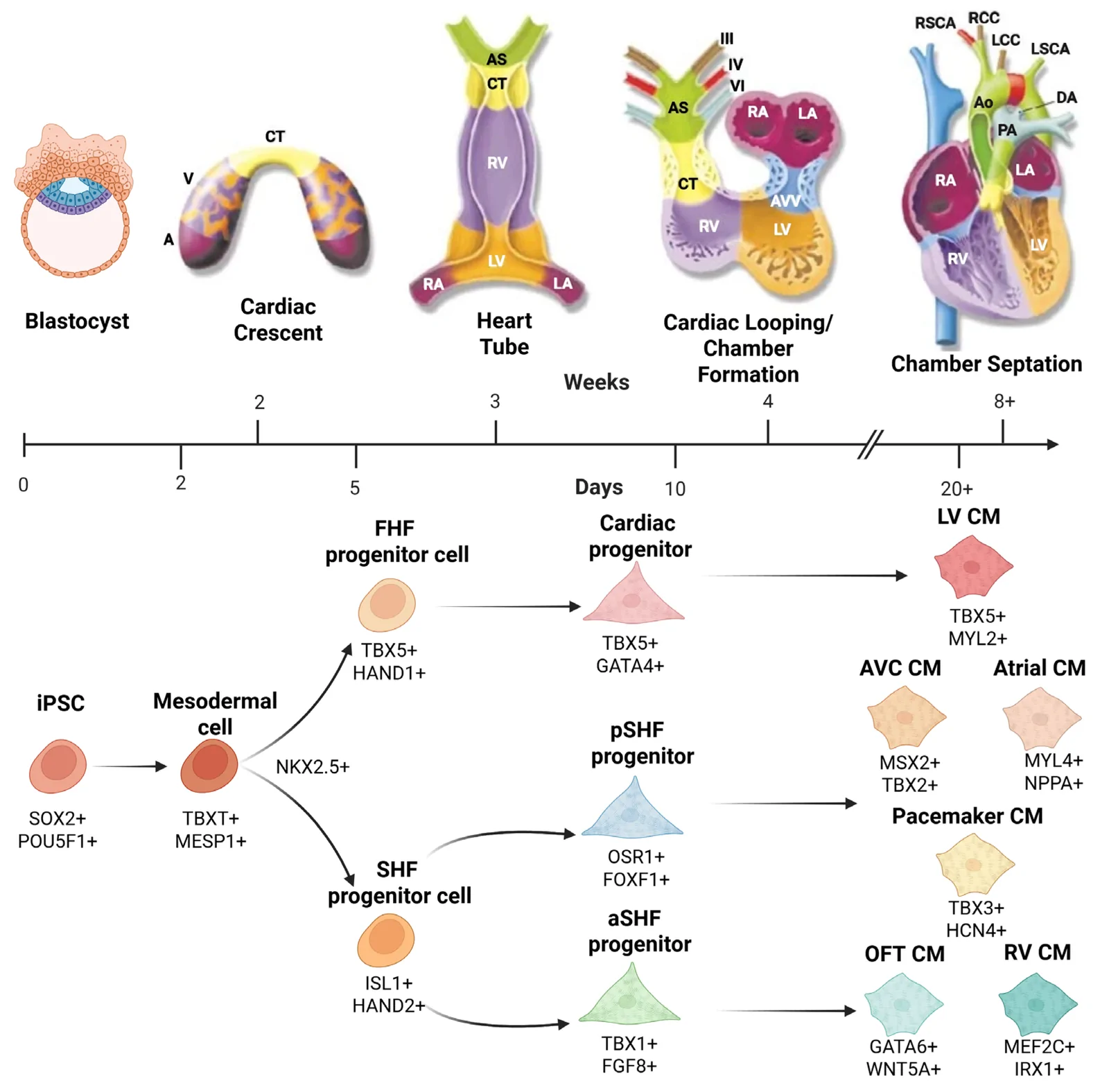
Cardiac morphogenesis and iPSC-CM differentiation. (**Top**) Developmental formation of a fully septated heart starting from fertilization at week 0 to cardiac crescent formation at week 2. A linear heart tube forms around week 3 and begins pulsating to provide circulation in the developing embryo. Cardiac looping and chamber specification starts around week 4, generating a fully septated heart around week 8. (A—atria; V—ventricle; CT—conotruncal; AS—aortic sac; RV—right ventricle; LV—left ventricle; RA—right atria; LA—left atria; AVV—atrioventricular valve; RSCA—right subclavian artery; RCC—right common carotid; LCC—left common carotid; LSCA—left subclavian artery; Ao—aorta; PA—pulmonary artery; DA—ductus arteriosus). (Adapted with permission from Srivastava & Olson [4] and Created in BioRender. Argall, A. (2026) https://BioRender.com/k3t5mw2). (**Bottom**) General iPSC cardiomyocyte differentiation denoting the generation of a common mesodermal progenitor, which can generate either FHF or SHF progenitor cells. FHF progenitors predominantly give rise to LV cardiomyocytes while SHF progenitors can form either posterior or anterior SHF structures. pSHF progenitors give rise to AVC, pacemaker, or atrial cardiomyocytes, while aSHF progenitors can form OFT or RV cardiomyocytes.

The heart is the first organ to develop in an embryo, the culmination of precise cellular and molecular events, many of which can lead to lasting negative consequences. Congenital heart disease remains the most common birth defect worldwide, and impacts nearly 1% of all live births in the United States [1]. Ventricular septal defects are the most common congenital heart defect (CHD). Major CHDs, including the tetralogy of Fallot, hypoplastic left heart syndrome, and pulmonary stenosis, share the characteristic of disease initiation during the first few weeks after conception. The heart is a distinctly organized structure derived from differentiation of cardiac mesoderm and progenitor cells. Each cardiac compartment is primarily derived from either the first heart field (FHF) or second heart field (SHF), with specific gene regulatory networks defining their boundaries. The FHF mainly generates the left ventricle (LV) and portions of the atria, whereas the SHF gives rise to the right ventricle (RV), outflow tract (OFT), atrioventricular canal (AVC), and part of the atria (Figure 1). The interventricular septum is a structure generated at the boundary between the FHF and SHF and is primarily driven by TBX5 expression [2]. Localized areas including the sinoatrial and atrioventricular nodes are derived by a complex gene network interaction between the FHF and SHF [3].

Induced pluripotent stem cells (iPSCs) can be reprogrammed from an individual’s somatic cells, providing a patient-specific genetic context for cellular development. Human iPSCs retain the genetic makeup of the individual they are derived from, and they can proliferate indefinitely and generate cells from the three germ layers [5,6]. Cardiomyocytes differentiated from patient-specific iPSCs can theoretically provide an unlimited resource for cardiovascular disease modeling and therapeutic development. Of note, iPSCs are inherently immature, and to date are unable to recapitulate a mature, adult cardiomyocyte phenotype. Further, due to ethical constraints, investigating human embryonic development is limited to model organisms. Delineating the “true” cardiomyocyte chamber identity from iPSC differentiations is relative to the gene expression detected in isolated human tissue and animal models, and even then, the signaling pathways and intergenic/exonic chromosomal regions may have limited similarities across species. Human iPSC-derived cardiomyocytes derived using the classic differentiation protocol (Wnt-ON/Wnt-OFF) often contain a mixed population of atrial, ventricular, and pacemaker-like subtypes without heart compartment-specific information. Recently developed protocols that sequentially administer growth factors and small chemicals to manipulate the key signaling pathways regulating the cell fate determination of the first and second heart field lineages during early heart morphogenesis have yielded compartment-specific cardiomyocytes (Table 1). These pathways include Wnt, BMP, Nodal, and Shh signaling, and can be manipulated in concert to differentiate human iPSCs into descendants of the FHF and SHF lineages. In this review, we focus on the recent development of protocols leveraged to generate compartment-specific 2D cardiomyocytes and 3D organoids from human iPSCs by the divergent induction of FHF and SHF lineages.

## 2. Modeling Human Heart Development Using iPSCs

**Figure 2:**
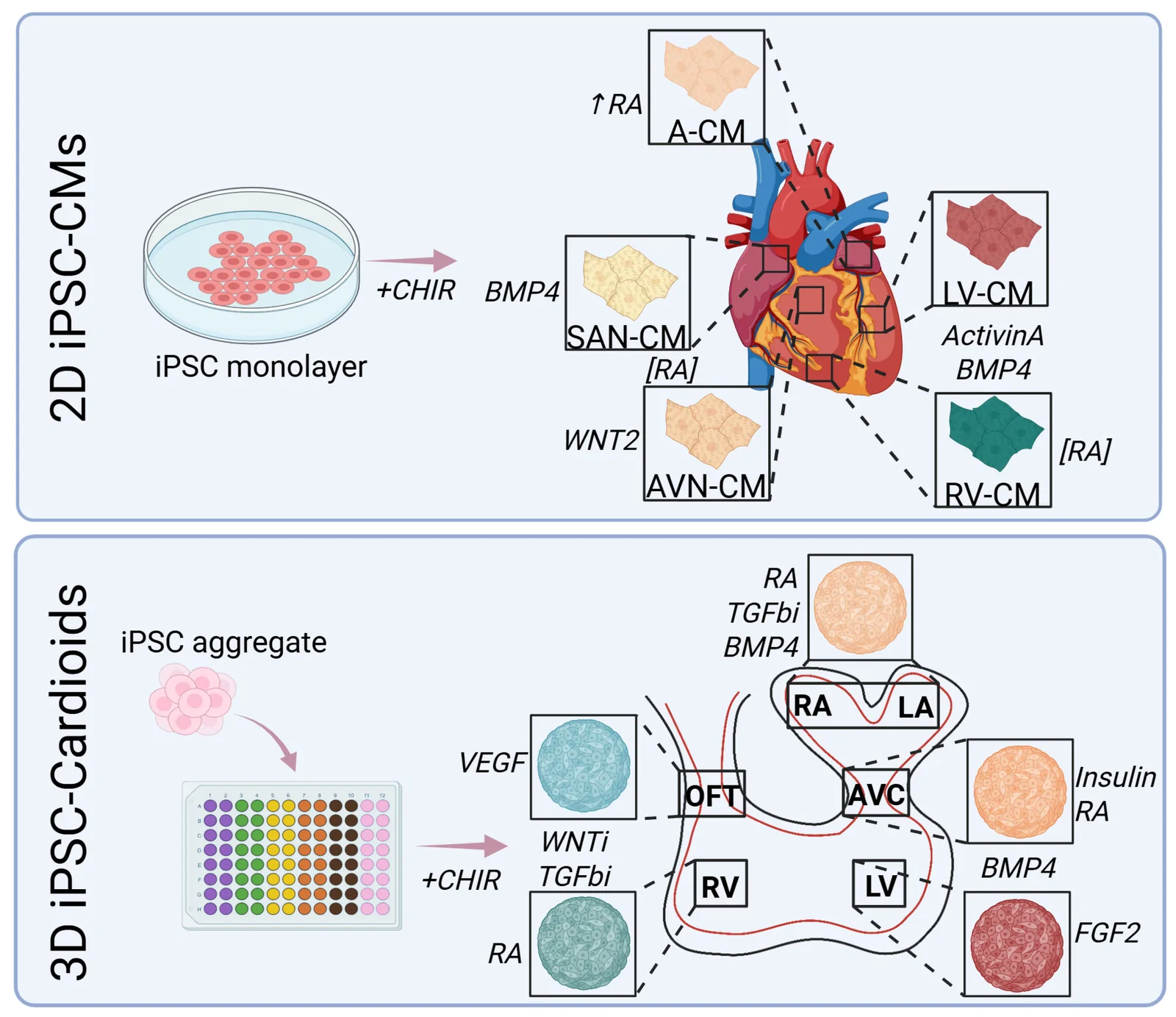
Compartment-specific iPSC-derived cardiomyocyte and organoid generation. (**Top**) Two-dimensional iPSC monolayer undergoing Wnt-ON/Wnt-OFF CHIR99021-mediated differentiation. While many of the compartment cardiomyocytes require similar signaling effectors to be generated, the molecules specific to each region are shown: A-CM requires specific concentrations of RA, RV-CM requires RA as well as Activin-A and BMP4, LV-CM is generated without RA, AVN-CM can be generated using Wnt ligands and RA, and SAN-CM requires BMP4 in addition to RA. (**Bottom**) Three-dimensional iPSC cardiac organoids (cardioids) undergoing CHIR99021-mediated differentiation. Compartment-specific organoids are either first generated by aggregating iPSCs in the beginning or later during differentiation, followed by treating them with specific compounds. The specific compounds to generate each compartment are as follows: OFT and RA require Wnt and TGFβ inhibition, while RV needs RA. Atria, AVC and LV all require BMP4, but LV needs FGF2, AVC needs insulin, and atria requires RA and TGFβ inhibition. Created in BioRender. Argall, A. (2026) https://BioRender.com/stg6hef.

Three weeks after fertilization, the primitive human heart tube begins beating and subsequently forms regions including the truncus arteriosus, bulbus cordis, primitive ventricle, primitive atrium, and sinus venosus. Each region of the heart is derived from the FHF or SHF of the cardiac crescent, generating specific cardiac compartments. The left ventricle forms from the primitive ventricle, whereas the right ventricle develops from the bulbus cordis [39]. The atria are formed from the primitive atrium and sinus venosus [11]. The SA node and the coronary sinus are derived from the sinus venosus, with the ascending aorta and pulmonary trunk forming from the truncus arteriosus [40]. Septation and valve morphogenesis occur during early cardiac development, with atrioventricular and semilunar valve formation continuing through the later stages of first-trimester development. From a cardiac crescent to a fully septated heart, the signaling effectors, including BMP, Wnt, FGF, Notch, and Shh, coordinate to pattern the heart with specific cardiac lineage cells. Modeling the major events during human cardiac development with human iPSCs can be achieved by adding the signaling effectors present in the embryo in a stepwise manner (Figure 2). An excellent detailed review has been published recently [41]. The temporal and spatial architecture of gene expression may misalign to what occurs in vivo, yet human iPSC-based cardiac differentiation still offers significant insight into disease-specific gene regulatory networks and cell-cell communication that otherwise might be obscured [42].

Although outside the scope of this review, engineered heart tissues (EHTs) have provided important evidence that cellular complexity improves the structural and functional maturation of human iPSC-derived cardiomyocytes. While many engineered tissues rely on the assembly of separately differentiated cell types within a scaffold or hydrogel, the organoid models discussed in this review are generated through the directed differentiation and self-organization of pluripotent stem cells into multicellular cardiac structures. Therefore, this review focuses on organoid approaches that attempt to model the developmental emergence of cardiac multicellularity rather than post hoc cellular assembly. In the following sections, we delve deeper into each cardiac compartment and how compartment-specific cardiomyocytes and organoids can be generated from human iPSCs. We also provide summary diagrams illustrating the protocols for generating compartment-specific cardiomyocytes from human iPSCs.

### 2.1. Ventricular Cardiomyocytes

#### 2.1.1. Left and Right Ventricular Development and Signaling Mechanisms

The left and right ventricles arise predominantly from spatially and temporally distinct populations of cardiac mesoderm, and much of what is known about their divergent origins derives from lineage-tracing and loss-of-function studies in mice, chicks, and zebrafish. During development, MESP1 marks multipotent progenitors common to both the FHF and SHF, and MESP1 dynamically regulates chromatin and gene expression patterns through ZIC2 and ZIC3 [43,44]. Further, a temporally distinct FOXA2+ population of cardiac progenitors, previously associated with endoderm and ectoderm lineage specification, contributes to both the FHF and SHF, as determined by murine lineage-tracing experiments [45,46]. The FHF, characterized by the expression of HAND1, TBX5, and HCN4, differentiates earlier and forms the cardiac crescent and contributes predominantly to the formation of left ventricular myocardium [47]. The basic helix-loop-helix (bHLH) transcription factor HAND1, when conditionally knocked out in αMHC or NKX2.5 expressing cells, results in ventricular malformations including ventricular septal defects, AV valve abnormalities and outflow tract abnormalities [7]. HAND1 is expressed in the outer curvatures of the embryonic LV post cardiac looping, while TBX5 expression is abundant within the cardiac crescent, and considerably higher in the primitive LV [8]. In contrast, the SHF, marked by the expression of ISL1, HAND2, PITX2, SIX1, FGF8, and FGF10, can be further classified as the anterior SHF (aSHF) expressing TBX1, SIX1, and FOXC2, which contribute to the RV cardiomyocytes, or the posterior SHF (pSHF) expressing NR2F2, HOXA1, and ALDH1A2, which contribute to the atrial cardiomyocytes [14,48].

Ventricular specification is coordinated by signaling pathways that balance progenitor expansion against differentiation in a stage-dependent manner. Canonical WNT/B-catenin signaling is biphasic: an early pulse from adjacent embryonic tissues promotes mesoderm induction and MESP1+ progenitor specification, whereas its later attenuation permits cardiac differentiation. This temporal logic has been demonstrated in zebrafish and mouse embryonic stem cells [49]. Within the SHF, sustained Wnt maintains progenitors in a proliferative state, with β-catenin required for ISL1 expression shown in mouse development [50,51]. Opposing this, BMP signaling promotes SHF differentiation as progenitors are incorporated into the right ventricle and outflow tract whereby loss of BMP type I receptor (BMPR1A) in ISL1-expressing cells caused abnormal right ventricular and outflow tract morphology and elevated ISL1 [52]. Retinoic acid then establishes anterior-posterior identity of the cardiac mesoderm. Loss of ALDH1A2 in mice resulted in the expansion of anterior SHF markers (TBX1, FGF8) posteriorly indicating that endogenous RA restricts SHF progenitors [27]. Following chamber specification, ventricular cardiomyocytes form both trabecular cardiomyocytes (finger-like folds extending into the lumen) under the control of Notch, Neuregulin, and Ephrins and compact cardiomyocytes through secreted factors from the epicardium and endocardium including Wnt, FGF, RA, and insulin-like growth factors [53,54,55,56,57,58].

#### 2.1.2. Generation of LV- and RV-like Cardiomyocytes

Human iPSC-derived ventricular-like cardiomyocytes can be generated under defined conditions through a Wnt-ON/Wnt-OFF protocol in which the GSK-3 inhibitor CHIR99021 prevents phosphorylation and degradation of β-catenin, allowing for β-catenin to translocate to the nucleus and facilitate a cardiac mesoderm transcriptional profile [59,60]. A few days later, Wnt signaling is turned off with the addition of IWR-1, a tankyrase inhibitor that interacts with axin, allowing for β-catenin phosphorylation and degradation, thus shutting off β-catenin mediated transcription [61]. Lineage tracing experiments using TBX5/MYL2-driven reporters suggest that the Wnt-ON/Wnt-OFF protocol primarily generates FHF-derived ventricular cardiomyocytes [62]. However, other studies using single-cell transcriptomics analysis indicate that FHF and SHF-derived cardiac cells co-exist in the population [63,64]. Therefore, it appears that classic Wnt-ON/Wnt-OFF protocol generates a mixed population of FHF and SHF derivatives, with ventricular-like cardiomyocytes as the predominate subtype alongside atrial and pacemaker cardiomyocyte SHF subpopulations [65] (Table 2).

**Figure 3:**
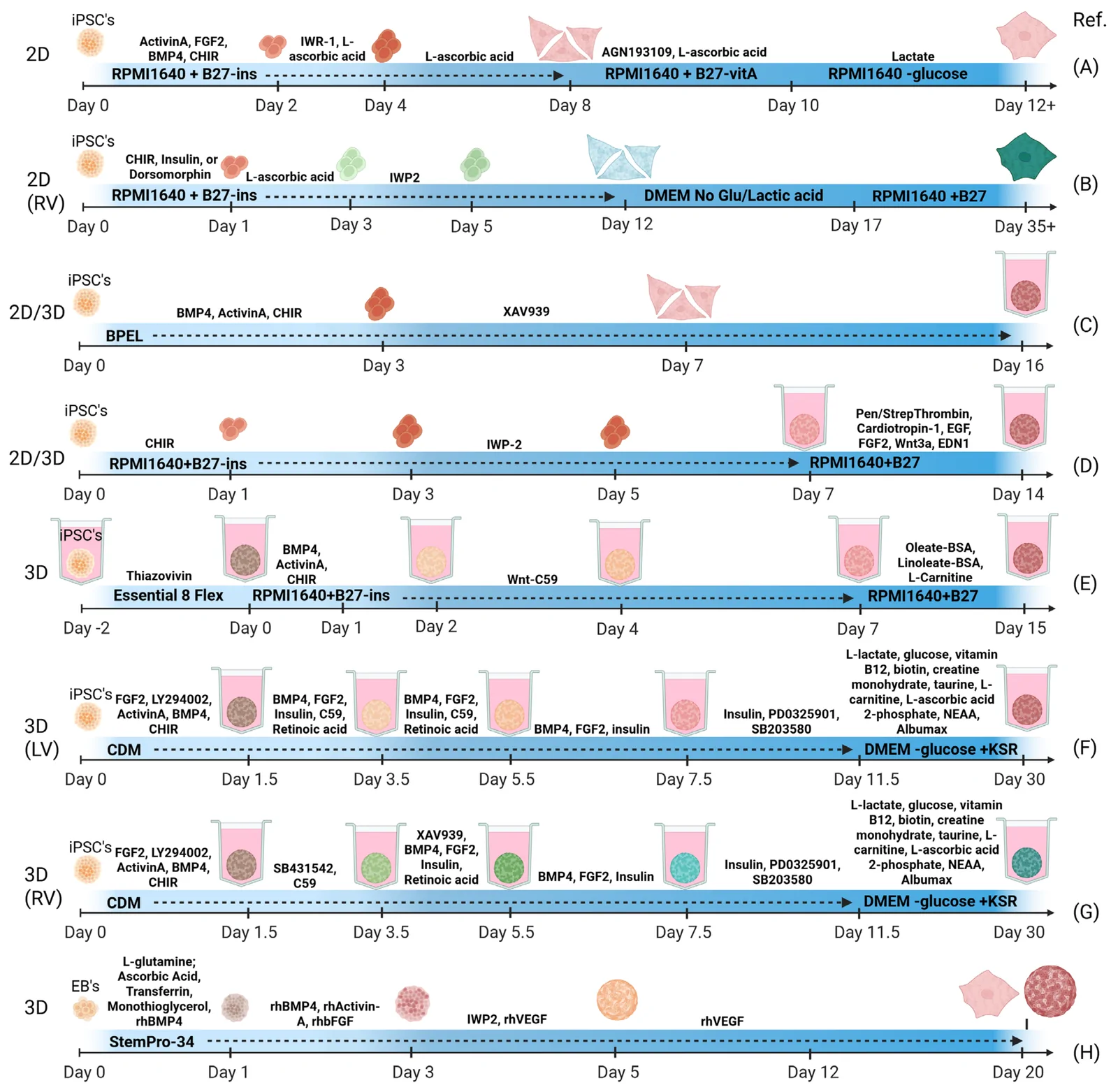
Ventricular cardiomyocyte differentiations. The protocols for 2D monolayer differentiation of iPSCs into ventricular cardiomyocytes vary. Many protocols that follow a Wnt-ON/Wnt-OFF paradigm generate a mixed population of cardiomyocytes, with the predominant cell type being ventricular cardiomyocytes, though some atrial and pacemaker-like cardiomyocytes are generated. Ventricular specifiers in the beginning stages of differentiation improve the generation of ventricular-like cardiomyocytes, specifically BMP4 and Activin-A. Distinct differentiation into left or right ventricular cardiomyocytes has been suggested through modulating the RA concentration, with low concentrations promoting right ventricle cardiomyocytes and high concentrations generating left ventricle cardiomyocytes, or by antagonizing BMP signaling to promote SHF gene expression. An organoid by itself denotes an EB aggregate differentiated towards cardiomyocytes, and an organoid within a media well denotes a cardiac organoid. Created in BioRender. Argall, A. (2026) https://BioRender.com/0bylsln. Refs.: (**A**) [66]; (**B**) [17]; (**C**) [68]; (**D**) [69]; (**E**) [70]; (**F**,**G**) [71]; (**H**) [72,73].

Recently Dark et al., improved the cellular phenotype of left ventricular cardiomyocytes by modulating the concentration of Wnt and adding ventricular specifiers (Activin A, BMP4) while inhibiting retinoic acid (RA) signaling (atrial inducer) with AGN193109, a pan-RA receptor inhibitor (Figure 3A) [66]. LV-specific iPSC-CMs generated through FHF progenitors exhibit increased metabolism and improved structural and functional maturity compared to age matched cardiomyocytes generated using standard Wnt-ON/Wnt-OFF protocol. RA is one driver of left vs right ventricular cardiomyocyte production: low RA concentration (0.05 μM) promotes right ventricular cardiomyocytes and higher concentration (0.1 μM) induces left ventricular cardiomyocytes from iPSCs [67]. By applying determinative concentrations of RA during a specific time window, LV- and RV-like cardiomyocytes and ultimately engineered heart tissues (EHTs) can be constructed with functional phenotypes similar to the LV and RV in the human heart. Concerning RV-like cardiomyocytes, Saito et al., determined that antagonizing BMP signaling using dorsomorphin or adding insulin during mesoderm induction of a Wnt-ON/Wnt-OFF protocol promoted SHF marker gene expression (FGF10, ISL1, CCK) and repression of FHF marker genes (TBX5, HCN4, HAND1) (Figure 3B) [17].

Protocol development spearheaded by Gordon Keller’s group and Sasha Mendjan’s group included generation of LV- and RV-specific cardiomyocytes and cardioids through induction of FHF and SHF cell lineages [56,71]. Keller’s group employed varying concentrations of BMP and Nodal signaling to push iPSCs toward FHF and SHF progenitors: a high Wnt/Nodal concentration induced FHF cell lineage differentiation, whereas a low BMP/Nodal concentration promoted SHF cell lineage differentiation (Figure 3H). Further, early administration of high RA concentration diverted the SHF into a posterior SHF cell lineage, primarily giving rise to the atria and atrioventricular canal (AVC), and late RA addition led to an anterior SHF, mainly contributing to the right ventricle and OFT. In parallel, Mendjan’s group leveraged Wnt and Activin signaling to divert the cell fate determination of FHF and SHF: low Activin/Wnt (stimulated by CHIR99021) determines the FHF’s cell fate, and high Activin/Wnt leads to the SHF’s cell fate determination. After mesoderm induction, low levels of RA and BMP with Wnt inhibition further drive FHF cell lineage differentiation. In contrast, maintaining a high RA concentration with dual inhibition of Wnt and TGFβ pathways directs the SHF into a pSHF cell lineage, and late administration of high RA concentration with Wnt inhibition directs the cardiac mesoderm towards aSHF. Overall, LV-like and RV-like cardiomyocytes can be generated by precise manipulation of Wnt/BMP/RA/Nodal signaling pathways through cell lineage divergence of the first and second heart fields. These LV-like and RV-like cardiac differentiation protocols can be applied to both 2D and 3D platforms, resulting in monolayer cardiomyocytes and 3D cardioids, respectively. As a result, the left ventricle (GJA1, HAND1, TMEM88, and TBX5) and right ventricle (IRX1, IRX2, and NPPB) markers are enriched in LV- and RV-like cardiomyocytes, respectively.

A similar chemical approach can be performed for 3D cardiac organoid differentiation, where iPSCs are first aggregated in a round-bottom well and co-differentiated over time (Figure 3E) [70]. This hybrid approach to generate a 3D cardiac organoid is conducted following a typical monolayer differentiation up to day 7, at which point the cells are aggregated in a round-bottom 96-well plate, then incubated with media containing chemical modifiers to promote a predominantly ventricular cardiac organoid, though other cell types are generated, including smooth muscle, endothelial, and epicardial cells (Figure 3D) [69].

Toward the goal of developing multi-chamber cardioids, a recent paper detailed the 3D generation of chamber specific cardioids including both left and right ventricular structures (Figure 3F,G) [71]. Higher RA and Activin-A concentrations generated RV-like cardioids compared to the concentrations used to generate LV-like cardioids, which contrasted with monolayer differentiation experiments, and suggested either the need for additional chemical modulators, like TGFβ inhibitors, to assist in right vs. left ventricular cardioid differentiation, or that the proximity of co-differentiating cells requires differential RA concentrations.

**Table 2:** Summary of ventricular-like cardiomyocyte differentiation protocols with an emphasis on differentiation efficiency, marker expression and phenotypic parameters.

Ref.	Cellular Model	Differentiation Efficiency	Functional Validation	Strengths and Limitations
[66]	2D hiPSC-derived left ventricular-like cardiomyocytes from H9 ESC line	Day 20 CM HAND1+/MYL2+ population reported at 91.9% efficiency via low Activin A, 5 ng/mL BMP4, and low CHIR (2–3 uM)	LVCMs expressed HAND1, TBX5, GATA4, TCAP, MYOM2, and TNNI3 (Day 20). Functional assays included electron microscopy to show longer sarcomeres compared to standard differentiation protocol, which is in line with mature cardiomyocytes; electrophysiology showed action potential amplitudes above 80 mV, with lower resting membrane potentials in part due to higher expression of KCNJ2 (*I*_K1_).	Strengths: Simple 2D differentiation protocol like standard Wnt-ON/Wnt-OFF paradigms that generates iPSC-CMs that mimic a left ventricular phenotype. Limitations: LVCMs still exhibited a maximum depolarization velocity similar to that of fetal cardiomyocytes and immature calcium release mechanisms—longer-term culture may improve maturation to a small degree.
[17]	2D hiPSC-derived right ventricular-like cardiomyocytes from either 201B7 iPSC line or HES3 ESC reporter line	Day 12 CM cTnT+ population reported at 70.4% efficiency with larger myocyte size using 1 µmol/L dorsomorphin	RVCMs expressed NKX2.5, FGF10, CCK, CXCR4, ISL1, MLC2v, and ANKRD1. Functional assays included electron microscopy showing poor sarcomere maturation; calcium transients in dorsomorphin-treated CMs had longer ‘time to peak’; electrophysiological analysis via patch clamping showed longer APD90 with reduced SCN5a expression.	Strengths: Chemical modulation approach that promotes SHF-like cardiomyocytes through the addition of a BMP antagonist during early cardiac development. Limitations: Uncertainty about a mixed population of cardiomyocytes generated during differentiation and transcriptional similarities to fetal human hearts via scRNAseq was not established.
[68]	2D ventricular cardiomyocytes generated using hiPSC line LUMC0099iCTRL#04	Day 20 CM TNNT2+ population reported at 77.6%	Ventricular CMs expressed ACTN2, NKX2.5, MYH7, MYL2 and SCN5a. Functional validation included electrophysiology showing prolonged cycle length, faster upstroke velocity, and longer APD20/50/90 times; carbachol treatment did not change cycle length but ivabradine reduced APD50/90.	Strengths: Robust electrophysiological characterization of ventricular hiPSC-CMs alongside recapitulation in a 3D context using engineered heart tissues. Limitations: Slight off-target generation of outflow tract cells using BMP4 and Activin-A.
[69]	2D-to-3D cardiac organoids generated using H7 ESC line	Day 7 FACS showed 50.1% SIRPα+ cardiomyocyte population	Ventricular cardiac organoids exhibited a mixed gene expression profile due to multiple cell populations, including SMA, CD31, TNNT2, and VCAM-1. Functional readouts included sarcomeric architecture and calcium transient recordings.	Strengths: Multicellular organoids containing both cardiomyocytes and cardiovascular progenitors shown to mimic cardiac hypertrophy phenotypes when stimulated with EDN1. Limitations: Did not investigate first heart field (left) vs. second heart field (right) ventricular identity of organoids. Implementation of the protocol requires FACS and precise mixing of CM and cardiovascular progenitors.
[70]	3D cardiac organoids generated with AICS-0037-172, iPSCORE_16_3, GCamp6f iPSC line, and hESC line H9	Day 14 organoids reported 64.9% TNNT2+ population using 4 uM CHIR	Organoids expressed HAND1, HAND2, MYL2, and MYL7, along with additional cell populations expressing NFATC1 and PECAM1. Functional validation demonstrated defined sarcomeres with neighboring mitochondria via transmission electron microscopy; microelectrode array and calcium imaging demonstrated normal action potential morphology and strong calcium waves.	Strengths: Robust chemical modulation of pluripotent stem cell aggregates to generate multicellular organoids. Transcriptomic profiles of the human heart organoids are closer to fetal hearts than monolayer differentiations. Limitations: No heart field specification, but both lineages are present. Mixed cardiomyocyte population with a predominating atria portion despite lack of RA—though quantification was performed via immunofluorescence.
[71]	3D LV and RV cardioids generated using hESC H9 line or WTC iPSC line	FHF-derived cardioids achieved a 95.4% TNNI1+ population and the aSHF-derived cardioids had an 88% TNNI1+ differentiation efficiency	LV cardioids expressed TBX5, NKX2-5, HAND1, and GATA4; RV cardioids expressed ISL1, FGF8, FGF10, FOXC2, TBX1 and IRX1. Functional validation for each cardioid type included transcriptomic comparison to in vivo heart dataset and mapping well to embryonic cell populations; electrophysiology via calcium transients showed LV having prolonged and faster transients (consistent with upregulation of GJA1) and RV cardioids having reduced contractions but long calcium bursts; field potentials via MEA showed LV having a consistent propagation field potential spread; and action potential acquisition via patch clamping exhibited diastolic potentials (~70 mV), upstroke velocity and amplitude of AP similar to that of other in vitro models. LV cardioids had high rates of spontaneous contractions (90%+) while RV had considerably lower rates (18%).	Strengths: Signaling-rich and comprehensive protocol that allows pluripotent cells to co-differentiate together in a heart field-specific manner that can be used to investigate compartment-specific heart defect development. Limitations: Only models early specification and morphogenesis and not later cardiac developmental stages, including heart looping, septation and valve formation. Complex media formulations may be a bottleneck for some labs, as vendor/batch variations can impact differentiation efficiencies.
[72,73]	2D ventricular cardiomyocytes generated from HES3 (reporter line with NKX2.5-GFP), H1 ESC and HES2 cell lines	95.1% CD235a/b+/ALDH- cells on Day 4, which generated ventricular-like CM on Days 12/20 with FHF gene expression when differentiated with 16 ng/mL of BMP4 and 8 ng/mL of Activin A	FHF-like CMs expressing TBX5, GJA1, and HAND1 on Day 20 with functional validation, including electrophysiology, where the FHF CMs exhibited on Day 35 prolonged APD50/90 compared to aSHF CMs, associated with the FHF CMs expressing higher levels of the KCND3 transient outward current (*I*_to_).	Strengths: Strong delineation between FHF and SHF chemical specifiers in a robust differentiation protocol. Limitations: Separating the heart field lineages into a distinct population requires FACS, though determining the specific concentrations of BMP4 and Activin-A can promote a specific heart field.

Interestingly, Schmidt et al. utilized the MEK inhibitor PD0325901 during the cardiac maturation phase of their cardioid differentiation, which developmentally instructed mesodermal cells to adopt a myocardial identity [71]. Since the inhibitor was added after cardiac specification, it should be interpreted as a maturation intervention rather than as instructing mesodermal cells to adopt a myocardial identity. This use differs from embryonic ventricular development, where MEK activity has been shown to act downstream of FGF but upstream of NKX factors to promote ventricular maintenance, while MEK inhibition reduces ventricular gene expression and permits ectopic atrial gene expression [74]. The investigators also temporally added low-dose RA earlier in the LV protocol, while the RV differentiation contained a short high-dose RA concentration that forced an aSHF commitment mirroring a critical developmental RA window needed for FHF-LV formation versus SHF-RV formation. Furthermore, the dose and timing of BMP4 and Activin-A in combination with Wnt activation, used in many protocols, has been suggested to direct differentiating cells between mesoderm and endoderm lineages, highlighting the need to modulate these parameters during early differentiation windows for optimal cardiac mesoderm induction [75]. Low-dose Activin-A with Wnt activation and inhibition can generate cardiomyocytes; although the proportion of ventricular to atrial cells was not quantified, this was leveraged to model left ventricular non-compaction from a patient with a KLHL26 variant and demonstrated disease-specific physiological and transcriptomic pathways [76]. Overall, ventricular cardiomyocyte differentiation has progressed from broadly generating ventricular-enriched cardiomyocytes to directing chamber-biased development through heart field specification. While canonical Wnt-ON/Wnt-OFF protocols generate ventricular-like cardiomyocytes, lineage-tracing and single-cell studies show that these cultures contain a mix of FHF and SHF cell populations. Aside from Wnt, approaches that modulate additional signaling pathways, including BMP, RA, TGFβ, and Nodal, can bias cardiomyocytes toward a ventricular identity. Importantly, none of these signals independently determine left or right ventricular identity, as their effect depends on the dose, timing, and pathway interactions within a 2D/3D differentiating environment.

### 2.2. AVC-like Cardiomyocytes

#### 2.2.1. AVC Development and Signaling Mechanisms

The atrioventricular canal (AVC) is a specialized region of the heart that plays a critical role in ensuring proper electrical and mechanical coordination between the atria and ventricles. AVC cardiomyocytes exhibit unique molecular and electrophysiological properties that distinguish them from other cardiac cell types. Understanding the developmental biology and signaling pathways involved in AVC formation has necessitated an interest in generating AVC-like cardiomyocytes from iPSCs. AVC formation is initiated by the establishment of a distinct myocardial domain, which is transcriptionally and functionally separate from the chamber myocardium. Notch signaling plays a pivotal role in this process by repressing chamber-specific gene expression while upregulating AVC-specific markers such as TBX2 and BMP2, ensuring that AVC cells maintain their unique identity [20,77,78,79].

Recent studies have provided compelling evidence that Wnt signaling is both necessary and sufficient for AVC patterning, reinforcing its role as a key determinant of AVC myocardial fate. In zebrafish, the loss of Wnt activity through axin1 overexpression led to the downregulation of bmp4 and tbx2b, resulting in the inappropriate expansion of chamber myocardial markers, such as nppa into the AVC region [22]. Moreover, Wnt functions upstream of BMP signaling to drive AVC formation. In apc mutant zebrafish, where Wnt/β-catenin signaling was ectopically activated, bmp4 and tbx2b expression expanded beyond the AVC into the chamber myocardium, reinforcing the role of Wnt in establishing AVC boundaries and preventing chamber-specific gene expression. Further, inhibition of BMP signaling in these Wnt-activated embryos abolished tbx2b expression, confirming that Wnt activity is mediated through BMP signaling to induce the AVC’s fate. These findings have established the Wnt–BMP–Tbx2 regulatory mechanism as a fundamental mechanism governing AVC specification. Studies in mice have provided further evidence supporting the role of Wnt signaling in AVC patterning and maintenance [80]. Using Axin2 reporter mice, Wnt activity was observed in the AVC from early embryonic stages (E10.5) through postnatal development. Loss of canonical Wnt signaling (Wnt LOF) led to a tricuspid atresia, hypoplastic right ventricle, and progressive loss of AVC myocardium, supporting its role in sustaining AVC identity. Conversely, ectopic activation of Wnt signaling (Wnt GOF) was sufficient to induce an AV junction-like phenotype in the ventricles characterized by myocardial constrictions; fibro-fatty deposition; and downregulation of chamber-specific markers, such as Scn5a and Cx43.

#### 2.2.2. Generation of AVC-Cardiomyocytes—2D/3D

**Figure 4:**
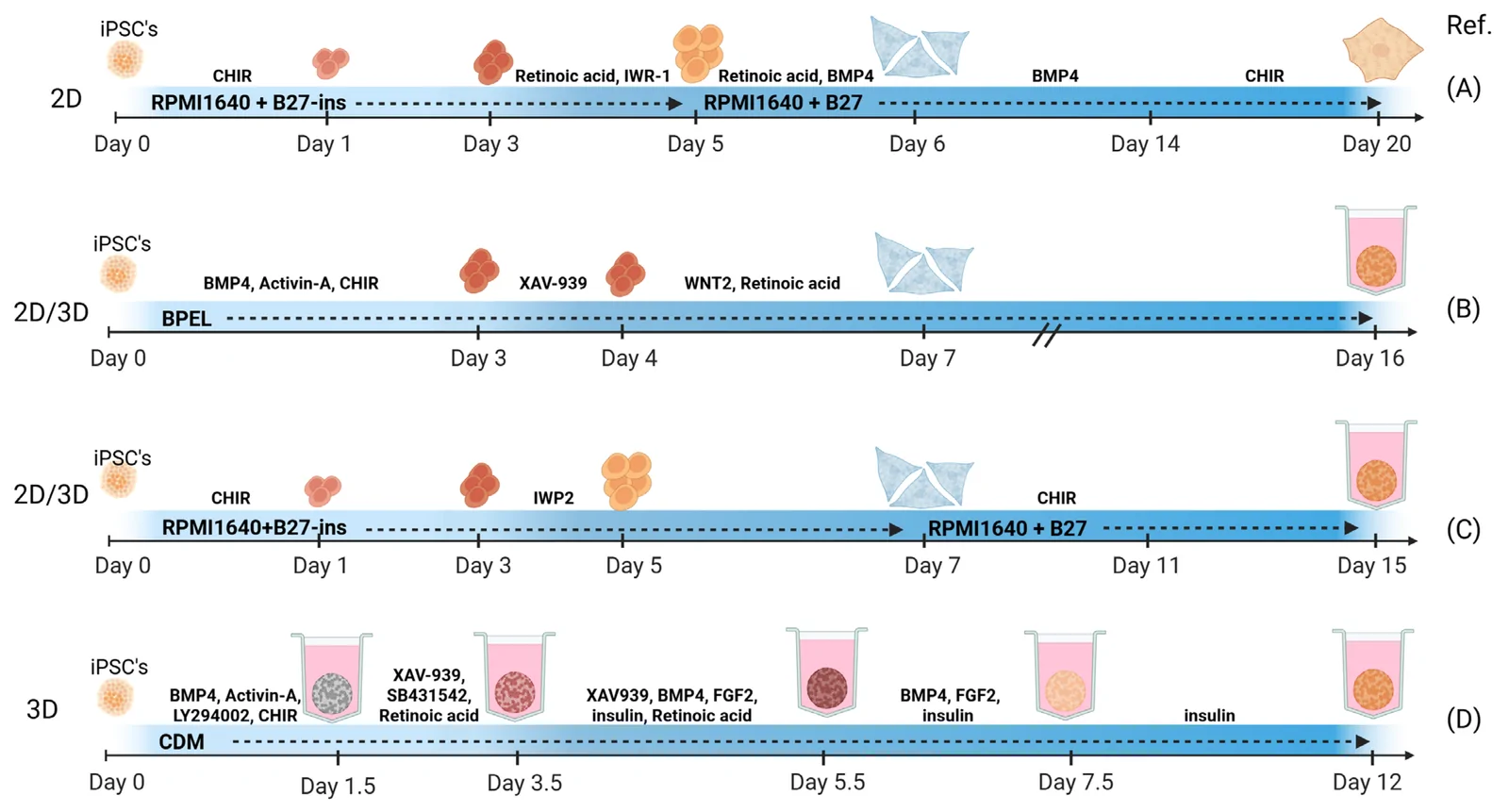
Atrioventricular canal (AVC) cardiomyocyte differentiation. Atrioventricular canal cardiomyocytes are generated though temporal exposure to both RA and a second stimulus of CHIR99021 to turn Wnt signaling back on. This specialized region, situated between the atria and ventricles, requires a mixture of signaling effectors to generate cells with transcriptomes comparable to those detected in vivo (e.g., BMP4 and Activin-A for ventricles, RA for atria). An organoid within a media well denotes a cardiac organoid. Created in BioRender. Argall, A. (2026) https://BioRender.com/r4zqkpi. Refs.: (**A**) [81]; (**B**) [82]; (**C**) [83]; (**D**) [71].

The development of atrioventricular canal-like cardiomyocytes (AVCLCs) from human iPSCs has seen significant advances through multiple differentiation strategies, signaling pathway modulations, and novel tissue-engineering approaches (Figure 4) (Table 3). Du et al. utilized a stage-specific differentiation protocol that modulated RA, Wnt, and BMP signaling to generate TBX3^+^/NKX2.5^+^/TBX18^−^/SHOX2^−^ AVCLCs (Figure 4A) [81]. RA was introduced between days 3 and 6, BMP4 from days 5 to 14, and Wnt activation using CHIR99021 from days 14 to 20. The resulting AVCLCs exhibited a low conduction velocity (0.07 ± 0.02 m/s) and relied on L-type calcium channels (*I*_Ca,L_) rather than sodium channels (*I*_Na_) for impulse conduction, reflecting their nodal-like properties. This stepwise approach enabled the controlled differentiation of homogeneous AVCLCs with well-defined molecular and electrophysiological characteristics, establishing a valuable foundation for generating conduction system cells in vitro.

Expanding beyond monolayer differentiation, Schmidt et al. introduced a cardioid-based organoid model, in which self-organizing human cardiac organoids (cardioids) were used to mimic atrial and ventricular chamber development alongside the AVC (Figure 4D) [71]. Unlike the directed differentiation approach of Du et al., this model facilitates the spontaneous emergence of FHF and SHF progenitors, which were organized into physiologically distinct cardiac compartments [81]. Single-cell RNA sequencing (scRNA-seq) and electrophysiology assays confirmed that AVC-like cardioids exhibited conduction delays akin to the AV node in vivo.

A unique biofabrication approach was developed by Chenxi Ye et al., who leveraged scRNA-seq and 3D bioprinting to generate AVC-like cardiomyocytes through the stage-specific activation of canonical Wnt signaling between days 11 and 15 (Figure 4C) [83]. The resulting cells expressed TBX2, MSX2, TBX3, and BMP4, mirroring the molecular signature of native AVC cardiomyocytes, and exhibited a distinctive small, round morphology. Notably, when incorporated into bioprinted “mini bow tie” cardiac tissues, these AVC-like cells functionally delayed electrical impulses, demonstrating their capacity to regulate conduction in engineered heart constructs. This study reinforced canonical Wnt signaling as a central regulator of AVC cardiomyocyte fate, and underscored the feasibility of integrating conduction cells into biofabricated heart tissues for regenerative applications.

Building upon these approaches, Jiuru Li et al. developed an assembloid-based system, integrating atrial, AVC-like, and ventricular spheroids to reconstruct the AV conduction axis in vitro (Figure 4B) [82]. This method employed WNT2 and RA signaling at the cardiac mesoderm stage to generate AVCMs that expressed the key markers TBX2, TBX3, MSX2, and BMP2. Optical mapping confirmed that the AVCM spheroids exhibited slower conduction velocities, mirroring physiological AV conduction delays, and replicating the fast–slow–fast activation pattern observed in vivo.

Although many AVC-like differentiation protocols were initially developed to establish molecular and electrophysiological identities, recent studies have begun to apply these systems to disease modeling. One example is the atrial–AVC–ventricular assembloid platform developed by Li et al. [82], in which patient-derived hiPSCs carrying an LMNA c.1477C>T variant and isogenic controls were used to model an LMNA-associated atrioventricular conduction block. By assembling atrial, AVCM, and ventricular spheroids in series, this system reproduced unidirectional fast–slow–fast conduction across the AV axis and identified abnormal intracellular calcium handling as a mechanism underlying AV conduction failure. Schmidt et al. used CRISPR-based perturbation of cardiac transcription factors, including TBX5 and FOXF1, to reveal compartment-specific defects in AVC and pSHF-associated differentiation [71]. More recently, CHD7-deficient multi-chamber organoids, modeling CHARGE syndrome-associated congenital heart disease, showed reduced TBX5 in ATR/AVC trajectories, decreased AVC marker TBX2, impaired cardiomyocyte differentiation, and abnormal contraction/calcium-transient behavior in AVC organoids [84]. Together, these studies indicate that AVC-like hPSC models could be potentially useful for studying AV conduction delay, AV block, and developmental defects involving atrioventricular septation, although patient-specific disease modeling remains limited to a small number of examples.

**Table 3:** Summary of atrioventricular canal-like cardiomyocyte differentiation protocols with an emphasis on differentiation efficiency, marker expression and phenotypic parameters.

Ref.	Cellular Model	Differentiation Efficiency	Functional Validation	Strengths and Limitations
[81]	2D hPSC-derived AV conduction-like cardiomyocytes generated from H7 hESCs.	Reported high AVC-like enrichment, with 90.7 ± 1.7% TBX3^+^/cTnT^+^ cells under the optimized AVCLC condition.	AVCLCs expressed TBX3, NKX2.5, TBX2, MSX2, BMP2, HCN4, and CX45, with low/absent TBX18, SHOX2, SCN5A, CX40, and CX43. Functional validation showed slow conduction velocity (0.07 ± 0.02 m/s) and preferential dependence on L-type Ca^2+^ current rather than sodium current.	Strengths: Simple and scalable 2D differentiation protocol for generating AV conduction-like cardiomyocytes with high reported TBX3^+^/cTnT^+^ enrichment. Limitations: Lacks 3D tissue architecture, multicellular chamber context, and spatial organization of the atrioventricular canal.
[71]	3D AVC-like cardioids generated within a multi-chamber cardioid platform from healthy hPSC lines. Functional data in Figure 4 were generated from WTC11 hPSCs; the study’s methods also listed use of H9 hESCs, WTC iPSCs, WTC11-derived Allen Institute reporter lines, and IMBA-derived control iPSC lines for broader validation/sex comparison work.	Not reported as a purified AVC-CM yield. No AVC-specific flow-cytometric yield or marker percentage output was reported.	AVC validation included pSHF/AVC marker expression (HOXB1, OSR1, TBX2, and TBX3), TBX2/TBX3 immunostaining and electrophysiological profiling. Functional assays included spontaneous contraction analysis, HCN4 RNA-seq, calcium channel and connexin profiling, GCaMP6f-based Ca^2+^ propagation, MEA field potential recordings, and patch-clamp analysis of dissociated AVC cardiomyocytes. AVC cardioids were reported to beat regularly.	Strengths: Multicompartment 3D human cardioid platform that models AVC-like development within a broader chamber-patterning context. The system captures pSHF/AVC-associated marker expression, multicellular organization, spontaneous beating, calcium signal propagation, MEA recordings, and patch-clamp analysis of AVC-like cardiomyocytes. Limitations: AVC population is not purified; differentiation efficiency is not reported; functional validation is tissue-level rather than focused on an isolated mature AVC/AV node cardiomyocyte subtype.
[83]	H1 & H9 hPSC-derived AVC-like cardiomyocytes incorporated into 3D bioprinted “mini bow tie” cardiac tissues; healthy/repository hESC model.	The protocol used a second CHIR99021 pulse from days 11 to 15 to induce an AVC-like fate in early cardiomyocytes. Day 15 flow analysis showed a dose-dependent decrease in the SIRPA^+^/CD90^−^ cardiomyocyte fraction with an increasing CHIR concentration: ~71.2% with DMSO, 69.3% with 2.5 μM, 52.1% with 5 μM, 37.4% with 7.5 μM, and 19.3% with 10 μM CHIR. The authors selected 5 μM CHIR as the optimal AVC-inducing condition because it preserved a substantial cardiomyocyte population while inducing AVC markers, with approximately 60% TBX2^+^/TNNT2^+^ and 45% MSX2^+^/TNNT2^+^ cells.	Molecular and lineage validation: Dose–response qPCR/relative expression for TBX2, MSX2, TBX3, BMP4, NKX2-5, SHOX2, ISL1, and TBX18; immunostaining for TNNT2/TBX2 and TNNT2/MSX2; scRNA-seq identified an AVCLCM cluster distinct from chamber-like cardiomyocytes and expressing AVC-associated genes; cross-species overlap analysis linked the human AVCLCM program to mouse AVC signatures. Electrophysiology/tissue function: Single-cell whole-cell current-clamp patch clamp paced at 1 Hz compared to DMSO chamber-like cells with 5 μM CHIR AVC-like cells using APA, MDP, APD50, APD90, and dV/dtmax readouts. Tissue-level function: In 3D bioprinted mini bow ties, optical mapping using calcium imaging showed a node delay phenotype only when the central region contained CHIR-induced AVC-like cells.	Strengths: Demonstrates that stage-specific canonical Wnt activation can induce AVC-like cardiomyocytes and that these cells can functionally delay electrical propagation when incorporated into spatially organized bioprinted cardiac tissues. The study provides tissue-level validation of AVC-like conduction delay and supports the use of engineered tissue geometry to model AV conduction. Limitations: The study does not provide one simple AVCM purity metric endpoint; the most compelling functional phenotype is demonstrated in a composite bioprinted tissue architecture, not as proof of a purified, mature AV node cardiomyocyte population in isolation.
[82]	3D assembloid of hiPSC-derived spheroids (atrial + AVCM + ventricular). Patient-derived hiPSCs with an AV-block LMNA variant (c.1477C>T) and isogenic control lines were used (plus healthy control iPSCs in parallel).	Not reported (no flow data or yield % given for AVCM).	Gene profiling: Cells in the WNT2 + RA (AV canal) condition expressed AV markers (TBX2, TBX3, MSX2, and BMP2). Optical mapping: The fused assembloid exhibited the expected fast–slow–fast conduction pattern across atrial–AV–ventricular regions.	Strengths: Provides the most physiologically integrated model of the AV conduction axis by assembling atrial, AVC, and ventricular spheroids into cardiac assembloids. The model recapitulates slow AVC conduction and a fast–slow–fast activation pattern. Limitations: The approach is technically complex and depends on reproducible generation and spatial assembly of multiple cardiac spheroid subtypes.

Collectively, AVC differentiation protocols are built on the developmental principle that atrioventricular canal identity represents a specialized, non-chamber myocardial program rather than an intermediate state between an atrial and ventricular myocardium. In vivo, an AVC myocardium is maintained through Wnt–BMP–TBX2/TBX3-associated repression of chamber myocardial genes and acquisition of slow-conduction properties. Current hPSC protocols model different aspects of this biology: Du et al. [81] used staged RA, BMP4, and late Wnt activation to enrich AV conduction-like cardiomyocytes in 2D; Ye et al. [83] demonstrated that a late canonical Wnt pulse can redirect early cardiomyocytes toward an AVC-like fate; Schmidt et al. [71] generated AVC-like cardioids within a broader pSHF-like 3D developmental context; and Li et al. [82] combined WNT2/RA-specified AVCMs with atrial and ventricular spheroids to reconstruct tissue-level AV conduction. Together, these models capture complementary aspects of AVC development, including molecular specification, suppression of chamber-like identity, and slow-conduction behavior. However, they remain partial models of AVC-like identity, as they do not fully recapitulate the spatial complexity, endocardial–myocardial interactions, cushion remodeling, or maturation events that shape AVC development in vivo.

### 2.3. OFT-like Cardiomyocytes

#### 2.3.1. Outflow Tract Development and Signaling Mechanisms

The outflow tract is a transient structure at the arterial pole of a developing heart. It initially forms a myocardial cylinder lined with endocardial cells before undergoing remodeling to establish the base of the ascending aorta and pulmonary trunk [85]. Development of the OFT involves the addition of progenitor cells from the SHF, a mesodermal population located within the pharyngeal region [16,86]. A defining feature of OFT development is the regionalized contribution of SHF progenitors. Lineage-tracing studies in mice have shown that the SHF is divided into anterior and posterior domains, each giving rise to distinct cardiac structures. aSHF progenitors contribute to the right ventricle and OFT myocardium, driving its elongation and eventual septation. In contrast, pSHF cells are involved in atrial and atrioventricular septation, primarily through the dorsal mesenchymal protrusion [48,87,88]. This spatial organization highlights the need for precise regulatory control, ensuring that progenitor populations are allocated correctly to form a functional heart.

The development of the OFT is orchestrated by the precise interplay of signaling pathways that regulate progenitor specification, proliferation, and differentiation. Among these, Wnt signaling plays a central role by modulating mesoderm induction and cardiac fate decisions [89,90]. Canonical and non-canonical Wnt signaling are essential for OFT formation, with early β-Catenin activation promoting mesoderm induction, while later inhibition facilitates aSHF specification and OFT differentiation. Fibroblast growth factors, especially FGF8 and FGF10, maintain SHF progenitors in a proliferative state and are required for OFT elongation, interacting with Wnt and BMP pathways to coordinate proper cardiac tube extension [15]. In addition to Wnt and FGF/BMP signaling, RA signaling plays a crucial role in regional SHF patterning. RA plays a compartmentalized role, where low levels promote aSHF identity and OFT formation, while exposure to high RA concentrations promote SHF progenitors and inhibits OFT specification [27].

#### 2.3.2. Generation of OFT-like Cardiomyocytes—2D/3D

**Figure 5:**
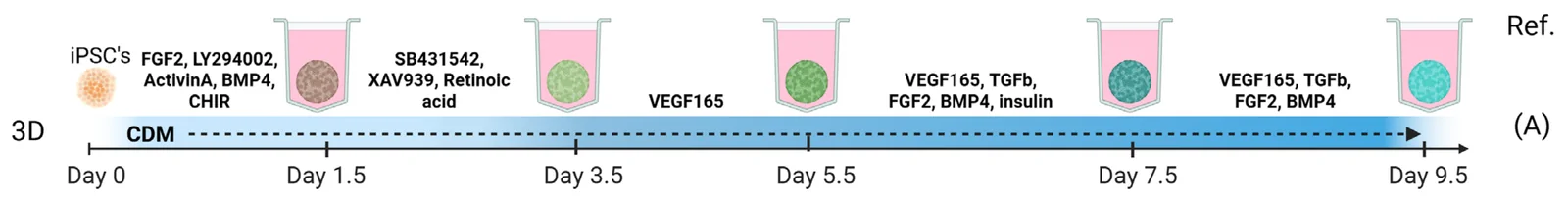
Outflow tract cardiomyocyte differentiation. To date, OFT cardiomyocytes have only been generated in a 3D format. Unique to OFT differentiation is the addition of vascular endothelial growth factor (VEGF) to facilitate the endothelial-to-mesenchymal transition. While the intent of this differentiation is to generate cardiomyocytes belonging to the OFT, a single-cell analysis has not been performed at various timepoints, so it is likely other cell populations are present, including valvular endothelial and interstitial cells. An organoid within a media well denotes a cardiac organoid. Created in BioRender. Argall, A. (2026) https://BioRender.com/4n5yzgn. Ref: (**A**) [71].

Recent advances in human cardioid platforms have enabled the in vitro modeling of the OFT by precisely modulating key developmental signaling pathways that govern progenitor specification (Figure 5) (Table 4). Schmidt et al. established a cardioid differentiation system that recapitulates OFT formation through a stepwise modulation of Wnt, Nodal/Activin, BMP, and RA signaling (Figure 5A) [71]. Wnt was activated to induce mesoderm, followed by canonical Wnt inhibition using XAV-939 to specify aSHF progenitors. BMP4 and RA were introduced during patterning stages to reinforce OFT identity. The resulting cardioids express key OFT markers including WNT5A, ISL1, HAND2, and RSPO3, closely resembling in vivo progenitors. Upon treatment with TGFβ and FGF2, cardioids undergo endothelial-to-mesenchymal transition, driving smooth muscle (ACTA2+) and endothelial (PECAM1+) differentiation. This system successfully recapitulates early OFT development, providing a model to investigate OFT development and disease.

OFT-like cardioid models have been applied less extensively to disease modeling than ventricular, atrial, or AVC systems, but emerging studies have indicated their value for studying anterior SHF-associated and conotruncal congenital defects. Schmidt et al. used the multi-chamber cardioid platform to model genetic and teratogen-induced perturbations affecting early heart compartment formation. In this system, ISL1-KO OFT cardioids showed reduced size, reduced cardiomyocyte differentiation, dysregulation of OFT-associated markers, impaired chamber formation, and a shift toward an atrial-like identity [26]. More recently, studies of CHD7-deficient multi-chamber organoids have extended this framework to CHARGE syndrome-associated congenital heart disease, a condition commonly associated with outflow tract and aortic arch abnormalities [84]. CHD7 loss suppressed the aSHF/OFT progenitor marker TBX1; produced an abnormal OFT morphology; and reduced OFT marker expression, including WNT5A/B. These studies have positioned OFT-like organoids as potentially useful models for early SHF patterning defects, conotruncal malformations, and developmental cardiotoxicity, although the current systems remain limited by their incomplete representation of cardiac neural crest, hemodynamic forces, and later OFT septation/remodeling.

OFT-directed differentiation is built around anterior second heart field patterning and arterial pole remodeling, rather than specification of a mature chamber-like cardiomyocyte subtype. In vivo, OFT formation requires the addition of aSHF progenitors to the arterial pole; coordinated Wnt, BMP, FGF, RA, Shh, and non-canonical Wnt signaling; and later interactions with endocardial, smooth muscle, and cardiac neural crest-derived populations. The current OFT-like cardioid protocol therefore emphasizes staged induction of aSHF/OFT progenitor identity and multicellular remodeling rather than purification of an OFT cardiomyocyte population. Schmidt et al. modeled this principle by using early mesoderm induction followed by Wnt/TGFβ modulation, RA/BMP/FGF signaling, and later VEGF/TGFβ/FGF-containing conditions to generate OFT-like cardioids enriched for WNT5A, ISL1, HAND2, and RSPO3 with endothelial and smooth muscle-like populations. This makes OFT cardioids useful for modeling early SHF patterning and arterial pole morphogenesis, but also highlights a major limitation: the current systems capture early OFT-like developmental states rather than later OFT septation, neural crest-dependent remodeling, hemodynamic forces, or mature outflow tract myocardium.

### 2.4. Atrial-like Cardiomyocytes

#### 2.4.1. Atrial Development and Signaling Mechanisms

The atria are the two upper chambers of the heart, which are divided into the left and right sides by a wall of tissue called the septum. The atria receive blood returning to the heart from the body and lungs and then pump blood down into the lower chambers to be circulated further. Understanding the developmental biology and signaling pathways involved in atria formation are important for generating atrial-like cardiomyocytes from iPSCs. Atrial development begins with the cardiac progenitor’s specification and the linear heart tube’s formation. As the heart undergoes looping, the atria emerge from the inflow tract through chamber ballooning, while PITX2 establishes left atrial identity. Subsequently, atrial septation separates the left and right atria through coordinated interactions between the primary atrial septum, endocardial cushions, and dorsal mesenchymal protrusion, ultimately forming the foramen ovale required for fetal circulation [91]. During atrial development, RA signaling is required and has an important role. RA signaling is mediated through the expression of RALDH2, which catalyzes the synthesis of RA in the posterior cardiac mesoderm adjacent to the venous pole [92]. Conversely, the CYP26 family of enzymes spatially restricts RA signaling through RA degradation, thereby maintaining anterior cardiac identities and ventricular specification [93]. Several transcription factors are enriched during atrial development including NR2F1, NR2F2, PITX2, TBX5, SHOX2, and GATA4, which collectively regulate atrial chamber morphogenesis, electrophysiological properties, and venous development [25,94]. Atrial cardiomyocytes acquire their electrophysiological and structural characteristics distinctly from ventricular cardiomyocytes during development. Atrial cardiomyocytes exhibit a shorter action potential duration, accelerated calcium-handling kinetics and distinct connexin expression profiles [95,96].

In mice, RA signaling specifies atrial identity by promoting atrial cardiomyocyte differentiation from progenitors in the pSHF during early heart development [27]. These RA levels are regulated by RALDH2, which synthesizes RA, and CYP26 enzymes, which degrade RA to establish the appropriate RA gradient required for cardiac patterning. After atrial specification happens by RA signaling between E7.5 and E8.5, atrial septation occurs between E10.5 and E13.5. During atrial septation, the primary atrial septum forms and fuses with the mesenchymal cap, vestibular spine, and endocardial cushions to separate the left and right atria.

In zebrafish, atrial septation does not occur because zebrafish possess a two-chambered heart. Instead of atrial septation, the cardiac disc elongates into a linear heart tube and loops to form distinct atrial and ventricular chambers by 48hpf. To do this, atrial progenitors are specified during gastrulation and migrate to the anterior lateral plate mesoderm. Also, RALDH2 synthesizes RA and restricts the specification of both atrial and ventricular progenitors, whereas subsequent BMP and Wnt signaling promote atrial cardiomyocyte differentiation [97].

#### 2.4.2. Generation of Atrial-like Cardiomyocytes—2D/3D

**Figure 6:**
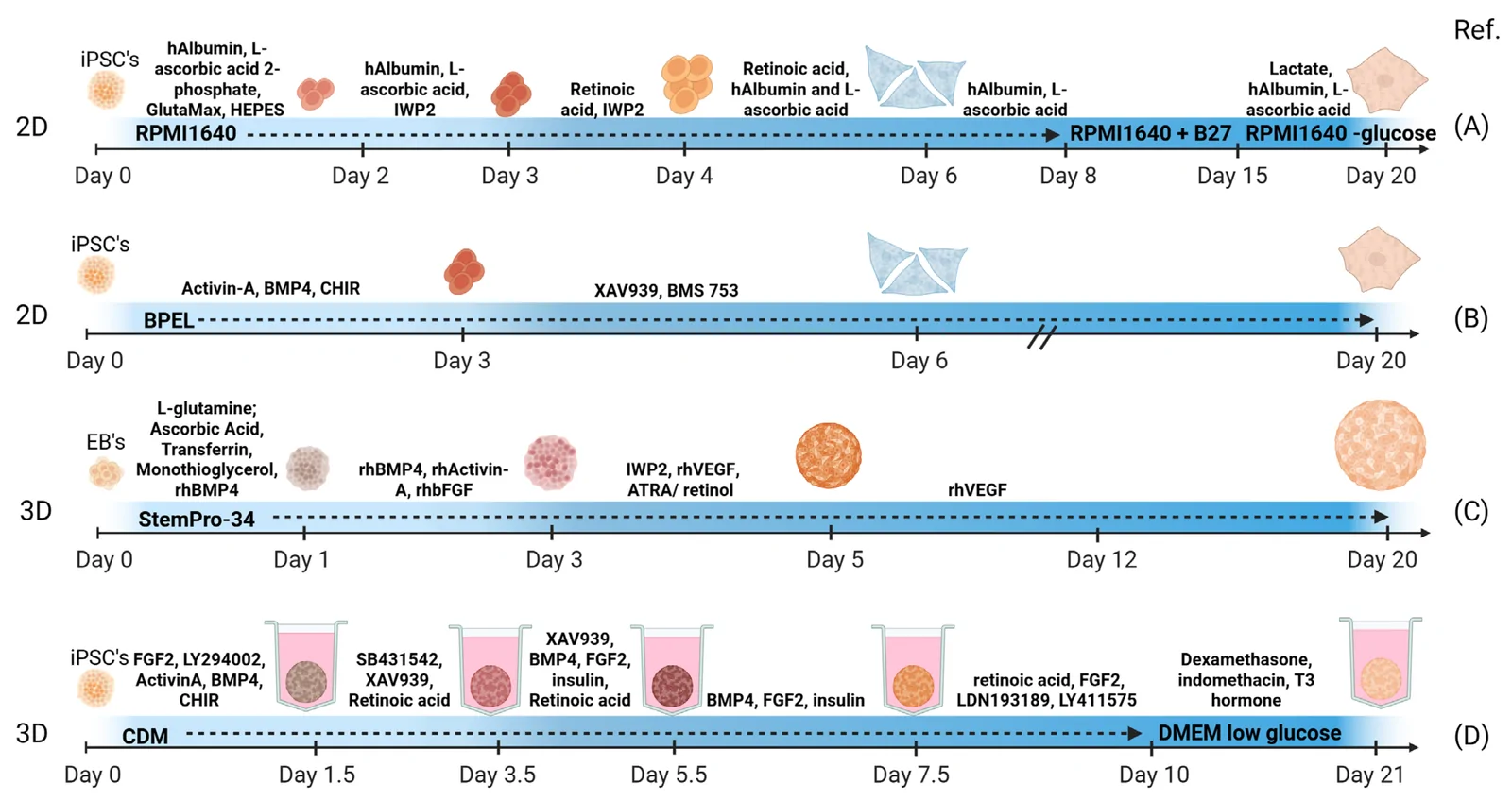
Atrial cardiomyocyte differentiation. Atrial cardiomyocytes are predominantly generated using RA, with the concentration and duration determining the atrial phenotype and efficiency of differentiation. To our knowledge, there have not been any protocols designed to generate left or right atrial cardiomyocytes and, as such, affords an area of opportunity. An organoid within a media well denotes a cardiac organoid. Created in BioRender. Argall, A. (2026) https://BioRender.com/mutnw3e. Refs.: (**A**) [100]; (**B**) [68,82]; (**C**) [72,73]; (**D**) [71].

Human iPSC derived atrial-like cardiomyocytes have been used to recapitulate chamber formation in the developing heart to investigate atrial cell signaling pathways [98], treat atrial fibrillation with atrial-selective drugs, and create engineered heart tissue [99]. Three routes of atrial-like cardiomyocyte generation have been developed: mesoderm induction, cardiac induction, and subtype initiation (Figure 6) (Table 5). Cyganek et al., established a robust protocol for generating hiPSC-derived atrial-like cardiomyocytes that closely resembled human atrial myocardium at the transcriptomic, proteomic, and functional levels. They also demonstrated that higher RA concentrations and prolonged RA exposure increased APD50, suggesting a shift toward a more mature atrial phenotype with distinct electrophysiological properties (Figure 6A) [100]. Li et al. generated hiPSC-derived atrial-like cardiomyocytes using retinoic acid-directed differentiation (Figure 6B) [68].

Li et al., generated hiPSC-derived atrial cardiomyocytes using BMS753 during subtype specification and confirmed atrial identity by the expression of atrial-specific genes and atrial-like electrophysiological properties (Figure 6B) [82]. Lee et al. generated atrial-like cardiomyocytes using an embryoid body-based differentiation system by exposing a cardiac mesoderm to RA during a defined developmental window. They demonstrated that atrial specification occurs specifically in the RALDH2^+^ mesoderm through autocrine RA signaling, leading to increased atrial gene expression and atrial-like electrophysiological properties (Figure 6C) [73]. Schmidt et al. recently described atrial-like cardioids generated from human iPSCs that resemble 3D heart structures (Figure 6D) [71]. Several steps are required to generate atrial-like cardioids: mesoderm induction, 3D cultures, patterning, cardiomyocyte specification, atrial chamber specification, and maturation. From day 0 to day 1.5 Actinin A, CHIR99021, FGF2, BMP4, and LY294002 promote differentiation into the pSHF-derived mesoderm, which gives rise to atrial-like cells. From days 3.5 to 5.5, XAV-939, BMP4, FGF2, insulin, and RA are added to drive atrial-specific differentiation. From days 5.5 to 7, BM4, FGF2, and insulin are added to promote further cardiomyocyte specification. From days 7 to 10, RA, FGF2, BMP inhibitor, and Notch inhibitor are added to enhance the atrial-like characteristics. From days 10 to 21, atrial cardioids are transferred to low-glucose DMEM containing dexamethasone, indomethacin, T3 hormone, and lipid concentrate to support long-term maturation [71]. RA signaling has a pivotal role in atrial-like cardioids as well as atrial-like cardiomyocytes.

In vivo, atrial identity is established during early cardiac development through RA signaling, which promotes atrial specification and the expression of atrial-associated genes. Current iPSC differentiation protocols have modeled this developmental process by modulating RA signaling during cardiac specification. Cyganek et al. applied 1 μM RA during days 3–6 after systematically optimizing the RA concentration and exposure duration to enrich atrial-like cardiomyocytes (Figure 6A) [100]; Li et al. used the RA receptor agonist BMS753 together with 5 μM XAV939 after cardiac mesoderm induction to generate atrial-like cardiomyocytes (Figure 6B) [68]; Li et al. treated cardiac mesoderm-derived progenitors with 1 μM BMS753 and 5 μM XAV939 for 72 h beginning on day 4 to specify atrial cardiomyocytes (Figure 6B) [82]; Lee et al. treated a cardiac mesoderm with 500 nM ATRA during days 3–5 and demonstrated that stage-specific RA signaling promotes atrial specification, particularly from an RALDH2^+^ mesoderm (Figure 6C) [73]; Schmidt et al. used 500 nM RA during pSHF patterning from days 1.5 to 5.5, followed by an additional RA treatment together with the BMP inhibitor LDN193189, and the Notch inhibitor LY411575 from days 7 to 10 to further promote atrial chamber specification and generate 3D atrial cardioids (Figure 6D) [71]. Together, these models capture complementary aspects of atrial cardiomyocyte development, ranging from RA-dependent lineage specification and chamber-specific gene expression to atrial-like electrophysiology and 3D cardioid organization. However, they remain partial models of atrial cardiomyocyte identity because most models do not fully reproduce the spatial complexity, multicellular interactions, left–right atrial patterning, or the progressive maturation of atrial myocardium in vivo.

Recent studies have incorporated atrial-like cardiomyocytes into engineered cardiac tissue platforms for chamber-specific functional assessments and disease modeling applications. Zhao et al. developed the Biowire II platform, a 3D engineered cardiac tissue system that incorporates atrial-like cardiomyocytes and cardiac fibroblasts within a hydrogel between two flexible polymer wires, enabling the electrical conditioning and functional assessment of atrial tissue [101]. This platform allows researchers to evaluate chamber-specific responses to pharmacological agents and model disease-associated phenotypes using patient-derived iPSC cardiomyocytes under long-term electrical conditioning. To demonstrate its application in disease modeling, the authors generated ventricular Biowires using iPSC-derived cardiomyocytes from hypertensive individuals with or without left ventricular hypertrophy (LVH). Long-term electrical conditioning was applied to mimic the chronic cardiac workload associated with hypertension, revealing LVH-associated changes in gene expression and impaired contractile function in the tissues derived from affected individuals. Reyat et al. generated iPSC-derived *PITX2*-deficient human atrial cardiomyocytes to investigate the links between *PITX2* deficiency, atrial cardiomyopathy, and atrial fibrillation (AF) [102]. They performed multiple assays on *PITX2*-deficient iPSC-derived atrial cardiomyocytes and found that a *PITX2* deficiency causes atrial mitochondrial dysfunction and a metabolic shift toward glycolysis in human atrial cardiomyocytes. *PITX2*-dependent metabolic changes may have contributed to the structural and functional defects found in the *PITX2*-deficient atrial cardiomyocytes. This study utilized an atrial cardiomyocyte differentiation protocol to demonstrate the potential of metabolic interventions as therapeutic strategies for preventing or treating *PITX2*-dependent atrial defects and AF.

### 2.5. Pacemaker-like Cardiomyocytes

#### 2.5.1. SA/AV Node Development and Signaling Mechanisms

Without input from the brain, the heart can generate and sustain beating due to the presence of specialized pacemaker regions, particularly the sinoatrial (SA) node and the atrioventricular (AV) node which serves as an electrical brake [103]. The SA node develops from a specialized region of the embryonic venous pole that is morphologically identifiable approximately 35 days into development [38,104]. Retinoic acid signaling promotes posterior SHF specification upstream of TBX5 [105], and TBX5 acts cooperatively with NKX2.5 to regulate SHOX2 and BMP4 expression during pacemaker region development [30]. SHOX2 represses NKX2.5, while ISL1 and TBX3 reinforce pacemaker identity by maintaining nodal gene expression and suppressing a working-myocardial program [31,34,36]. TBX18, but not NKX2.5, is expressed within the myocardium of the sinus venous and SA node, with the expression of HCN4 and TBX3 delineated by PITX2C [3,32].

The AV node develops from a specialized region in the dorsal region of the AVC and is recognizable at approximately 5 weeks of gestation [106,107]. Localized BMP2-SMAD signaling establishes AVC identity by inducing TBX2 and MSX2, preventing the myocardium from adopting a chamber identity [21]. TBX2 and TBX3 participate in a feed-forward interaction with BMP2 and TGFβ2 that maintains the AVC myocardium and coordinates endocardial cushion development [19]. AVC conduction is much slower than that of the atrial and ventricular chambers, due in part to the expression of Cx30.2; this region is also characterized by the expression of GJC1/Cx45, HCN4, and TBX3 and lacks the expression of GJA5/Cx40, GJA1/Cx43, or SCN5A/Na_v_1.5 [108,109,110]. Unlike the SA node, the AV node expresses TBX2 and MSX2 [111]. These two pacemaker regions function to precisely control cardiac conduction within the atria and throughout the ventricular myocardium [112].

#### 2.5.2. Generation of Pacemaker-like Cardiomyocytes—2D/3D

**Figure 7:**
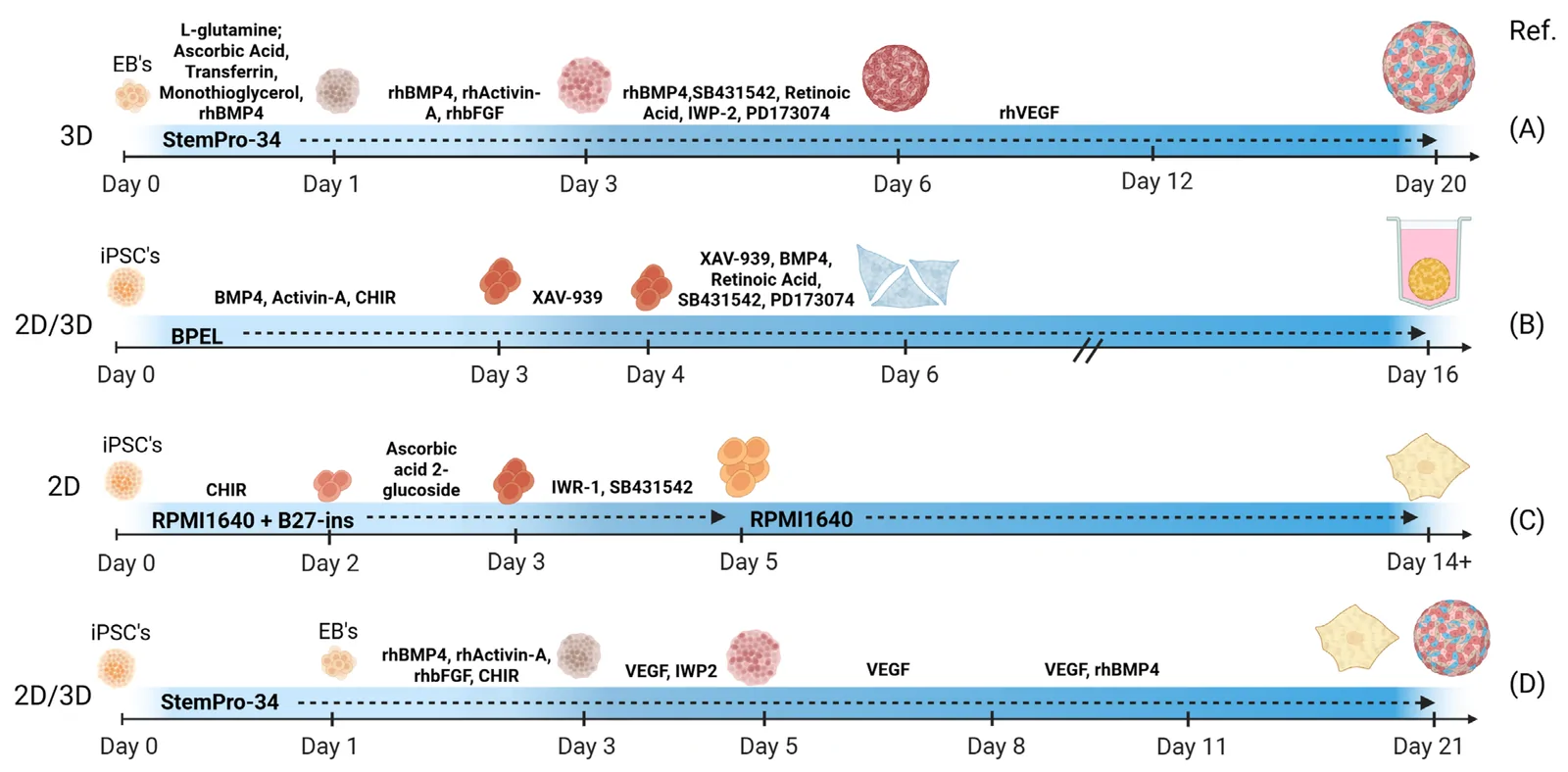
Pacemaker cardiomyocyte differentiation. Cellular automaticity, or being able to generate an action potential, is a defining feature of pacemaker cardiomyocytes. Pacemaker cardiomyocytes derived from iPSCs share aspects of atrial differentiation due to the use of RA when concerning the SA node, while one approach for the AV node excludes RA. An organoid by itself denotes an EB aggregate differentiated towards cardiomyocytes, and an organoid within a media well denotes a cardiac organoid. Created in BioRender. Argall, A. (2026) https://BioRender.com/1ju60fb. Refs.: (**A**) [35]; (**B**) [68,82,113]; (**C**) [114]; (**D**) [115].

Generation of pacemaker-like cardiomyocytes holds great promise in the field of regenerative medicine; one such approach is to generate these cells from a patient’s own cells for reimplantation for the treatment of conduction defects. Recapitulating what is known to occur in vivo, many groups have developed in vitro 2D and 3D methods to generate pacemaker-like cardiomyocytes expressing SHOX2 and HCN4 through the modulation of Wnt, RA, BMP, and Nodal signaling (Table 6). SA node-like pacemaker cells have been generated from iPSCs upon sequential exposure to BMP; Activin-A; RA; and inhibitors of FGF, TGFβ, and Wnt (Figure 7A) [35,113]. These SA node-like pacemaker cells express the markers of the SA node, including TBX18, SHOX2, and TBX3; however, it has also been found that these cells co-express NKX2.5, previously found to be absent in murine and human studies, which may suggest retention of cardiomyocytes with chamber-like identity [33]. SA node cardiomyocytes from the head or transitional zone regions have also been generated through either late-stage inhibition of Wnt or early addition of TGFβ2, respectively (Figure 7B) [113].

Another strategy utilizes overexpression of known transcription factors (e.g., TBX3, TBX18) responsible for the formation of pacemaker cells. By rewiring differentiating cardiac progenitors with pacemaker-specific transcription factors, iPSC cardiomyocytes can recapitulate a nodal-like phenotype with spontaneous depolarization and decreased SCN5A (Nav1.5), GJA1 (connexin-43), and KCNJ2 (Kir2.1) expression [116,117]. Overexpression of HCN2 is also shown to increase the production of pacemaker-like cardiomyocytes from iPSCs, whereas increasing the expression of HCN4 only improves the pacing ability of the cardiomyocytes through enhancement of the *I*_f_ pacemaker current [118,119]. PITX2C is known to inhibit SA node development in vivo with the downregulation of SHOX2 expression; in response, Nodal inhibition using SB431542 between days 3 and 5 of cardiac mesoderm differentiation of iPSCs increases HCN4+/HCN1+/TNNT2+ cardiomyocytes (Figure 7C) [114]. AV node-like pacemaker cells have recently been developed through similar approaches to the generation of AVCLCs with a Wnt-ON/Wnt-OFF/Wnt-ON stimulation paradigm along with BMP (Figure 7D) [115]. Lohbihler et al. demonstrated that AV node-like pacemaker cells resemble the transcriptomic profile of fetal core AV node pacemaker cells, as well as exhibiting comparable conduction velocities to a human AV node. Unique to this differentiation protocol is the lack of RA present; the authors noted the low expression of RALDH2, a marker of RA-dependent progenitors, in the mesoderm population, explaining why they excluded it from their protocol. Collectively, these studies underscore the potential of gene overexpression and pathway inhibition to promote the generation of pacemaker-like cardiomyocytes.

One nuance of pacemaker-like cardiomyocyte differentiations that requires further optimization is the use of either VEGF or SB431542, an ALK5 inhibitor, during the Wnt inhibition stage. VEGF is likely functioning to maintain a VEGFR2/KDR+ progenitor pool rather than trying to specify pacemaker identity. While TGFβ signaling may not alter myocardial populations [113], it may promote a nodal-like transcriptional profile by reducing Nodal-dependent PITX2C expression and relieving repression of the SHOX2-TBX3 signaling pathway. These contributions again are time- and dose-dependent, as evident by the ability to generate head, transitional zone, and tail-like SAN cardiomyocytes. Recently, modulation of the AMPK pathway via the activator AICAR or inhibitor Compound C (Dorsomorphin) was shown to enrich SA node-like cardiomyocytes post Wnt inhibition, allowing for the study of sick sinus syndrome [120]. Collectively, developmental signaling approaches can establish regionally defined SA or AV node identities, while transcription factors and ion channel overexpression strategies can introduce selected pacemaker properties. Marker expression and spontaneous automaticity alone are therefore insufficient to confirm nodal identity and must be coupled with an electrophysiological analysis, including the action potential morphology, conduction properties, and autonomic responsiveness.

## 3. Future Applications and Approaches

**Figure 8:**
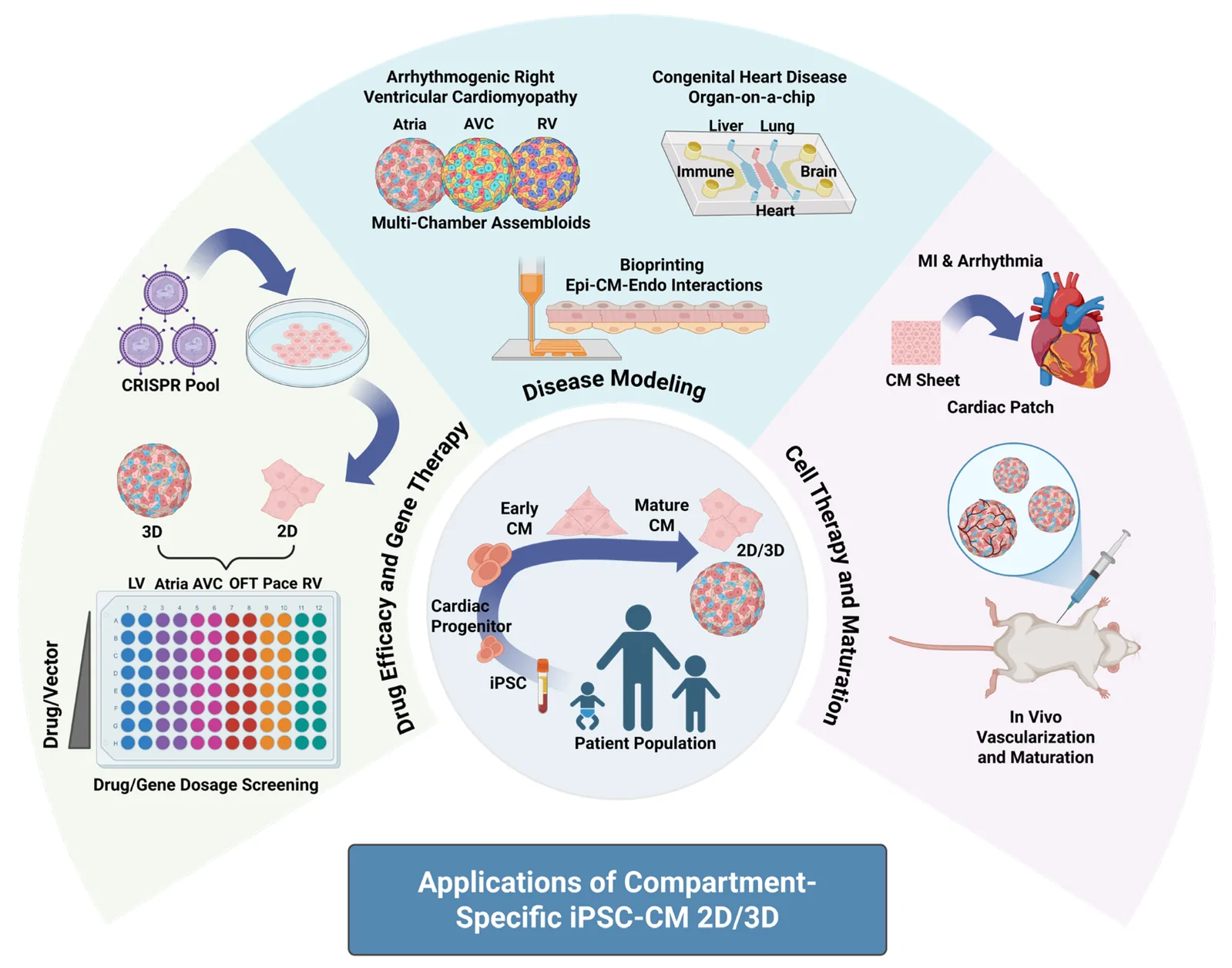
Applications of compartment-specific iPSC-CMs in 2D and 3D. Drug Efficacy and Gene Therapy: Usage of a CRISPR pool to knockdown/out specific genes or introduce novel mutations to test their impact within each cardiac compartment as either a monolayer or organoid. Further drug screening for novel pathway modulators to promote cardiac maturation could be implemented for each cardiac compartment. Disease Modeling: Assembloids consisting of multiple organoids from each compartment to mimic cardiac conduction within the heart to test various diseases associated with arrhythmia and cardiomyopathies. Organ-on-a-chip technology for monolayer cells to assess secreted signaling crosstalk between various organ-specific cell types. Bioprinting epithelial, myocardial, and endothelial cell layers to investigate cell–cell interactions. Cell Therapy and Maturation: Cardiac patch creation for transplantation onto hearts impacted by a myocardial infarction or to bypass arrhythmia circuits. Implantation of organoids into model organisms for vascularization and maturation, possibly with the intent to repair cardiac function. Created in BioRender. Argall, A. (2026) https://BioRender.com/bfpclnv.

The previous sections have summarized recent advances in the methodologies for generating compartment-specific cardiomyocytes and cardiac organoids that recapitulate distinct regions of the developing and mature heart. Building upon these developmental strategies, the following section highlights the current and emerging applications of these models for testing drug efficacy and gene therapy, modeling specific diseases, and usage for cell therapy and in vivo maturation (Figure 8). The ability to reprogram patient-derived somatic cells in iPSCs and subsequently drive their differentiation into highly specialized cardiac cell types represents a major paradigm shift in cardiovascular biology and precision medicine. Advances in compartment-specific differentiation and multicellular organoid systems provide opportunities to model regionally restricted cardiac phenotypes, cell–cell interactions, and developmental signaling networks with increasing physiological relevance. A recent detailed review highlights the bioengineering applications of promoting these concepts [121].

### 3.1. Bioengineering-Guided Disease Modeling

The predominant application of these iPSC-derived chamber-specific cardiomyocytes lies within the biomedical engineering space, with the creation of organ-on-a-chip technologies that can allow for crosstalk between several cell types. iPSC-derived cardiomyocytes, fibroblasts, and vascular endothelial cells have been seeded into the microchannels of a chip, generating ventricular cardiomyocytes with stronger IRX4 gene expression and contractility, more accurately mimicking the in vivo behavior of ventricular cardiomyocytes [122]. Primarily used as 2D monolayers, this application can also incorporate iPSC-derived cell populations from a patient to study cellular-level organ crosstalk, e.g., studying the signals secreted from cardiomyocytes derived from a single ventricle in a patient with a disease; studying the relationship between hepatocytes and alveolar cells to assess fibrosis; or to assess neuronal dysfunction within a brain compartment or macrophage responses with an immune component [123]. Other modalities could incorporate electrical stimulation and fluid dynamics to better mimic the in vivo condition [124].

Assembloids are complex structures consisting of different types of organoids physically connected to assess a specific function. Recently, an assembloid comprising an atrial, an AVC, and a ventricular organoid connected in series showed that the AVC organoid slowed conduction from the atrial to the ventricular organoid; additionally, this assembloid modeled an AV block by assessing loss of function in the presence of an LMNA mutation [82]. Other applications include generating assembloids from patients with arrhythmogenic right ventricular cardiomyopathy to assess conduction deficits to the right ventricular organoid or to assess arrhythmia circuits from patients with atrial fibrillation. While not exclusive to cardiac compartments, others have coupled cardiac organoids with an immune component such as macrophages [125]; or produced tri-tissue assembloids consisting of skeletal, muscular, and neural organoids to study the neuromusculoskeletal axis during development [126]. An excellent review highlights other cardiac organoid applications for disease modeling and drug screening [127].

### 3.2. Drug Efficacy and Gene Therapy

The development of compartment-specific cardiomyocytes allows for the testing of drugs to assess cell-type specific consequences for the treatment of diseases, particularly cardiotoxic effects of chemotherapeutics or screening novel agonists/antagonists that may promote cardiac maturation or differentiation in a particular compartment. Usage of multiple cell populations in 3D tissue-like structures including iPSC-derived cardiomyocytes, endothelial cells, and fibroblasts, has been shown to provide high sensitivity to a drug screen for arrhythmic-inducing compounds [128]. Compartment-specific iPSC cardiomyocytes can be used to screen potential gene therapy targets and small-molecule compounds important to cardiac function. Using both CRISPR interference and activation along with small-molecule screening, iPSC cardiomyocytes have identified causal genes and potential therapeutic targets when applied to doxorubicin-induced cardiomyopathy [129]. The readouts for cardiotoxicity in iPSC-derived cardiomyocytes can include cell death or even excitotoxicity though calcium imaging when administering various drugs that influence ion channels [130]. Additionally, genetic perturbations during cardiac differentiation can also be dissected with CRISPR screens to identify cardiac-specific regulators [131].

### 3.3. Cell Therapy and Maturation

Translational applications of compartment-specific cardiomyocytes and organoids may be relevant when generating a cardiac sheet for implantation onto a heart following myocardial infarction, or to bypass an arrhythmic circuit. These patches can integrate with the host tissue, provide structural support, and enhance cardiac function [132,133]. Furthermore, injection of pacemaker-like cardiomyocytes into a cryoablated guinea pig heart has been shown to maintain AV node conduction in vivo and provides a framework for a biological conduction bridge between the atria and ventricles in the context of AV block [115]. Implantation of cardiac organoids into a host organism provides an in vivo paradigm to allow an organoid to both mature and become perfused by the host’s circulatory system. In vivo implantation near a vascular-rich area has been shown to promote maturation, although to date this has only been shown using lung, intestine, and colon organoids, affording opportunities to expand on these findings in the cardiac field [134].

### 3.4. Limitations to iPSC-CM and Cardiac Organoids

Despite substantial progress in generating compartment-specific cardiomyocytes and cardiac organoids from human pluripotent stem cells, several limitations exist that restrict their interpretation and translational applications. One major challenge is cellular immaturity. Many hiPSC-derived cardiomyocytes retain fetal-like electrophysiological, metabolic, and structural features. While this immature state may be appropriate for disease modeling of cardiac developmental and congenital defects, this limits the ability to study diseases that emerge in mature cardiomyocytes with adult-like phenotypes. Therefore, compartment identity should not be inferred from marker expression alone, but supported by functional readouts, including action potential morphology, calcium handling, contractility, and metabolic state. A second challenge is reproducibility. Compartment-specific differentiation protocols depend on narrow windows of Wnt, BMP, FGF, Nodal, retinoic acid, Notch, and Shh signaling modulation. Small differences in the stem cell line background, passage number, cell density, reagent lot, modulation timing, and endpoint analysis can alter the lineage composition and maturation state. These issues are amplified in cardiac organoids, where self-organization introduces additional variability in size, spatial patterning, and cellular composition. Single-cell and spatial transcriptomic benchmarking against human embryonic and fetal cardiac reference datasets will also be important for distinguishing true compartment specification from transitional developmental states.

Current disease models also have important limitations. Patient-specific iPSC models preserve the genetic background of the donor, but this strength can confound interpretation when comparing unrelated control and diseased lines. Isogenic gene-edited controls for specific genes or chromosomes can reduce this variability, but may not fully recapitulate complex genetic heart disease. Many organoid models represent early cardiac development and may not capture later events, including valve maturation, innervation, or long-term maturation. These limitations are important in the context of congenital heart diseases that arise from multiple cardiac lineages. Future progress will require integrating developmental biology with bioengineering and robust functional validation. For compartment-specific models, directed lineage induction with controlled multicellular patterning will be required, while organoids will need to maintain their advantage of self-organization while improving reproducibility and functional readouts. Studies modeling diseases should incorporate multiple patient lines; consider clonal variability; generate isogenic controls if possible and assess longitudinal readouts during differentiation, including transcriptomic, structural and physiological assays. Together, these advances will help shift compartment-specific iPSC-derived cardiomyocytes and organoids from transcriptionally descriptive models towards mechanistically rigorous platforms for studying cardiogenesis and modeling chamber-specific cardiac pathology.

## 4. Conclusions

Compartment-specific cardiac cell populations allow for more precise modeling of congenital heart diseases impacting a specific area of the heart. In this review, we summarized the developmental origin and signaling pathways of each compartment to provide a biological context for how investigators have modeled in vitro differentiation studies. The recent 2D and 3D differentiation strategies relevant to cardiac compartments highlight the growing ability to direct pluripotent stem cells toward left ventricular, right ventricular, atrial, atrioventricular canal, outflow tract, and pacemaker/node-like identities through the temporal modulation of signaling pathways. Despite the rapid progress, the current models are limited by immaturity and variability across PSC lines. Compartment-specific iPSC-derived cardiomyocytes and cardiac organoids offer a powerful framework for studying cardiovascular diseases and, as such, require protocols to improve maturation, increase physiological relevance, and reduce differentiation variability. The goal of this review was to synthesize recent methodologies to help inform future investigations on how best to differentiate iPSCs into compartment-specific cardiomyocytes while providing a contextual framework for how these cells can be used to study cardiovascular disease.

## Figures and Tables

**Table 1:** Inferred developmental origins, representative molecular markers, and expected functional properties of major cardiac compartments. Functional properties describe mature or lineage-associated electrophysiological and contractile tendencies, these may be immature, incomplete, or context-dependent in embryonic tissues, organoids and hiPSCs.

Compartment	Inferred Developmental Origin	Markers/Marker Identity	Expected Functional Features
Left Ventricle	First heart field predominant myocardial lineage (in vivo lineage tracing studies)	HAND1 [7], TBX5 [8], MYL2/MLC2v [9], IRX4 [10], NKX2-5 [11], TNNT2 [12], MYH7 [13]	Fast *I*_Na_-driven upstroke, stable resting potential (~−85 mV), long action potential duration with sustained *I*_Ca,L_-mediated plateau phase, high conduction velocity (Cx43 rich), robust SR-dependent calcium cycling (large calcium transient amplitude, fast uptake via SERCA2a), high contractile force generation.
Right Ventricle	Anterior second heart field predominant myocardial lineage	ISL1 [14], TBX1 [14], FGF8 [15], FGF10 [16], FOXC2 [17], HAND2 [7], IRX3 [18], IRX1 [18]	Fast *I*_Na_-driven upstroke, stable resting potential (~−85 mV), shorter APD than LV with more prominent early repolarization (phase 1 notch, higher Ito density), high conduction velocity, robust but slightly smaller calcium transients relative to LV, lower wall stress and contractile force output.
Atrioventricular Canal	Atrioventricular canal myocardium	TBX2 [19,20], MSX2 [21], BMP2 [21,22]	Slow, *I*_Ca,L_-dependent upstroke, low conduction velocity (Cx45 dominant, low Cx43), slow-type action potential with no sustained plateau, intrinsic automaticity possible, minimal contractile output; functionally critical for establishing AV conduction delay prior to formation of the mature conduction system.
Outflow Tract	Anterior second heart field derived OFT myocardium with cardiac neural crest, endocardial and smooth muscle cell contributions during arterial pole remodeling	ISL1 [14], TBX1 [14], WNT5A [23], GATA6 [24], SEMA3C [24]	Slow, *I*_Ca,L_-dependent upstroke, primitive/transitional action potential morphology, slow conduction velocity, minimal contractile function; smooth muscle cell and neural crest contributions yield a mixed excitable phenotype during arterial pole remodeling that is lost as the OFT myocardium remodels into the subpulmonary conus and aortic root.
Atria	Posterior second heart field/inflow-tract associated myocardial lineage	NR2F2/COUP-TFII [25], NPPA [26], ALDH1A2 [27], KCNA5 [28], MYL4 [9], KCNJ3 [28], GJA5 [29]	Fast *I*_Na_-driven upstroke, stable resting potential (~−80 mV), short triangular APD with minimal plateau (prominent *I*_Kur_, *I*_KACh_), high conduction velocity (Cx40/GJA5 rich), smaller calcium transient amplitude relative to ventricle (sparser t-tubule network, lower SR calcium load), lower contractile force.
Sinoatrial Node	Right horn of the sinus venosus/venous pole-derived pacemaker lineage	SHOX2 [30,31], TBX18 [32,33], ISL1 [34], HCN4, CACNA1D [35]	No stable resting potential, spontaneous diastolic depolarization driven by the coupled-clock mechanism (membrane clock: *I*_f_/HCN4, *I*_CaT_; calcium clock: rhythmic SR calcium release via RyR2), slow *I*_Ca,L_-dependent upstroke, low conduction velocity (low Cx43/Cx40 coupling with nodal-type connexin profile), minimal contractile function; primary pacemaker activity.
Atrioventricular Node	Dorsal atrioventricular canal-derived conduction lineage	TBX3 [36], TBX2 [19], GATA4 [37], ISL1 [34], HCN4 [38], HCN1 [28]	Slow, *I*_Ca,L_-dependent upstroke, spontaneous diastolic depolarization (latent/backup pacemaker), low conduction velocity with decremental conduction properties (Cx45 dominant), no sustained plateau, minimal contractile output; functionally responsible for physiological AV delay and subsidiary pacemaker function.

**Table 4:** Summary of outflow tract-like cardiomyocyte differentiation protocols with an emphasis on differentiation efficiency, marker expression and phenotypic parameters.

Ref.	Cellular Model	Differentiation Efficiency	Functional Validation	Strengths and Limitations
[71]	Healthy hPSC-derived 3D OFT-like cardioids; primarily generated using WTC/WTC11 iPSCs and WTC11-derived reporter lines	OFT identity was primarily assessed by marker enrichment. Functionally, only a small fraction of OFT cardioids showed spontaneous contraction, indicating limited contractile maturation relative to LV, atrial, and AVC cardioids.	Validation supported an OFT-like developmental and multicellular tissue identity, evidenced by OFT-associated marker expression (WNT5A, ISL1, HAND2, and RSPO3) and emergence of endothelial and smooth muscle-like populations (PECAM1, ACTA2).	Strengths: Provides an early human 3D platform for modeling OFT-like development in a multicellular context; captures OFT-associated marker expression together with endothelial and smooth muscle-like cell populations relevant to arterial pole remodeling. Limitations: Not a purified OFT-CM protocol; limited OFT-specific electrophysiological maturation.

**Table 5:** Summary of atrial-like cardiomyocyte differentiation protocols with an emphasis on differentiation efficiency, marker expression and phenotypic parameters.

Ref.	Cellular Model	Differentiation Efficiency	Functional Validation	Strengths and Limitations
[100]	Healthy hPSC-derived 2D atrial-like cardiomyocytes; primarily generated using dermal fibroblasts, mesenchymal stem cells, and peripheral blood mononuclear cells	Stage-specific 1 μM RA treatment (days 3–6) generated 87.1 ± 2.5% cTnT^+^ CMs, with 94.7 ± 0.8% MLC2V^−^/MLC2A^+^ atrial cardiomyocytes	ACMs expressed atrial markers MYL7 (MLC2A), NR2F2, NPPA, KCNA5, CACNA1D, GJA5 (Cx40), HEY1, TBX5, and MYH6, while ventricular markers MYL2, GJA1, and IRX4 were reduced.	Strengths: Established a highly reproducible monolayer protocol with high atrial purity (~95% MLC2V^−^/MLC2A^+^). Limitations: The model is limited to 2D atrial cardiomyocytes and does not recapitulate tissue-level atrial architecture or multicellular interactions.
[68]	2D hiPSC-derived atrial cardiomyocytes by RA-mediated directed differentiation	Directed differentiation produced 70–80% TNNT2^+^ cardiomyocytes	ACMs expressed atrial markers NPPA, NR2F2, and KCNJ3, while ventricular marker MYL2 remained low. Functional assays included single-cell RNA-seq and patch clamp (atrial-like action potentials, negative resting membrane potential, and rapid spontaneous beating).	Strengths: Simple protocol. Provides comprehensive molecular and electrophysiological characterization of atrial cardiomyocytes. Limitations: The model is limited to 2D atrial cardiomyocytes.
[82]	3D assembloid of hiPSC-derived spheroids (atrial + AVCM + ventricular); patient-derived hiPSCs with an AV-block LMNA variant (c.1477C>T) and isogenic control lines were used (plus healthy control iPSCs in parallel)	Approximately 79.1% TNNT2^+^ cardiomyocytes	ACMs expressed atrial markers NPPA, NR2F2, MYL7, and MYL4. Functional assays included flow cytometry, RT-qPCR, scRNA-seq, immunostaining, patch-clamp electrophysiology, and optical mapping.	Strengths: Demonstrated that chamber-specific atrial cardiomyocytes can be reproducibly generated and incorporated into multicellular cardiac assembloids, enabling tissue-level studies of atrial physiology and electrical conduction. Limitations: Atrial cardiomyocytes served mainly as a reference/control population for AV canal modeling; therefore, atrial differentiation and maturation were not extensively optimized or characterized.
[73]	hPSCs–3D embryoid body derived from 2D atrial cardiomyocytes	Control cells generated 87% NKX2-5^+^/cTnT^+^ and 63% MLC2V^+^/cTnT^+^; Day 3 RA generated 92% NKX2-5^+^/cTnT^+^ and 2% MLC2V^+^/cTnT^+^	ACMs expressed NR2F2 (COUP-TFII), NPPA, MYL7, TBX5, KCNJ3, KCNA5, CACNA1D, and GJA5, with reduced MYL2, IRX4, and MYH7. Functional assays included flow cytometry, immunostaining, qRT-PCR, calcium imaging, and patch-clamp electrophysiology, demonstrating atrial-like action potentials and electrophysiological properties.	Strengths: Demonstrated that stage-specific RA signaling acting on RALDH2^+^ cardiac mesoderm directs atrial lineage specification. Limitations: Atrial differentiation efficiency was not directly quantified using atrial-specific markers. The embryoid body-based protocol is relatively labor-intensive, with greater batch-to-batch variability.
[30]	3D atrial cardioids generated within the multi-chamber cardioid platform from healthy hPSC lines; functional data in Figure 4 were generated in WTC11 hPSCs; the study methods also list H9 hESCs, WTC iPSCs, WTC11-derived Allen Institute reporter lines, and IMBA-derived control iPSC lines for broader validation/sex comparison work	Efficient cardiac differentiation produced >85% TNNI1^+^ cardiomyocytes	Atrial cardioids expressed NR2F1, NR2F2, TBX5, and HEY1. Functional assays included RNA-seq, scRNA-seq, immunostaining, calcium imaging, optical mapping, multi-electrode array (MEA), and patch-clamp electrophysiology.	Strengths: Established the first 3D chamber-specific atrial cardioid generated from posterior second heart field progenitors, closely recapitulating early human atrial development and chamber-specific electrophysiology. Limitations: The atrial cardioids resemble early fetal atrial tissue rather than mature adult atria.

**Table 6:** Summary of pacemaker-like cardiomyocyte differentiation protocols with an emphasis on differentiation efficiency, marker expression and phenotypic parameters.

Ref.	Cellular Model	Differentiation Efficiency	Functional Validation	Strengths and Limitations
[35]	Embryoid body-based differentiations using HES3-NKX2.5-GFP reporter line, HES2, and MSC-IPS1 lines	Day 20 SA node-like pacemaker cells generated around 35% NKX2.5-/cTNT+ cells when exposed to low levels of BMP4 (2.5 ng/mL) on Day 3 of differentiation.	Day 20 expression denoted via TBX5, SHOX2, ISL1, TBX18, HCN1, and HCN4, with low MYL2. Electrophysiological validation showed Day 20 SANLPCs exhibited fast spontaneous firing rates, slow maximum upstroke velocities, small AP amplitudes and short AP durations. Higher densities of *I*_f_ and *I*_KACh_ currents were present and the calcium current density was higher (*I*_CaT_). Cells responded accordingly to beta-adrenergic and muscarinic stimulations.	Strengths: Protocol allows for pacemaker-specific differentiation that is transgene-independent. Limitations: In vivo studies demonstrate immature-like beating rates of SANPLCs and differentiation protocol results in a mixed cellular population, suggesting chemical optimization is required per line.
[113]	LUMC0099iCTRL#04 and LUMC0047iCTRL#1A hiPSC lines were used to generate 2D SANCMs and EHTs	Day 20 SANCMs generated anywhere from 79.9% to 88.5% TNNT2+ cells.	Day 20 SANCM expression included TBX18, SHOX2, HCN4, and TBX3. Functional validation with electrophysiology showed SANCMs had a lower AP amplitude, slower upstroke velocity, short APD20/50/90 and less negative maximum diastolic potential. Funny current blockage with ivabradine specifically increased cycle length in SANCMs while ventricular CMs were unaffected.	Strengths: Noted that the SANCMs’ electrophysiological parameters mimicked those of freshly isolated human SA nodes. scRNAseq transcriptomic comparison demonstrated that cells of SA node’s tail, head, and transitional zone can be generated with this protocol. Limitations: SANCMs are relatively still immature
[114]	HiPSC lines iPS-DF19-9-7T and iPSC-DF6-9-9T from WiCell were used to generate pacemaker-like CMs	Day 14 hiPSC-CMs expressing TNNT2 generated between 28.2% and 44.4% cells when treated with IWR1 and SB431542 between Days 3 and 5.	Day 20 hiPSC-CM expressed KCNA5, HCN1, HCN4, TBX18, TBX3, and SHOX2. Electrophysiology validation was performed on advanced hiPSC-CM cultures past Day 40 and exhibited less negative maximum diastolic potential and shorter APD50/90.	Strengths: An iPSC-based differentiation protocol that modulates Nodal signaling instead of multiple signaling pathways. Limitations: Low efficiency for generating a homogeneous population of SA node-like cardiomyocytes with immature electrophysiological parameters.
[115]	HES3-NKX2.5-GFP; TBX3-tdTomato and HES2 ESC from WiCell and ESI-017-ASAP1 line	Day 20 AV node-like pacemaker cells (AVNLPC) expressing SIRPA+/CD90+ were generated with 12 uM CHIR at 79.5% efficiency or 87% when stimulated with BMP2 between Days 8 and 11.	Day 20 AVNLPCs expressed MSX2, TBX2, and TBX3. Functional validation via electrophysiology in engineered AVN tissues showed faster diastolic depolarization, slower maximum action potential upstroke velocities and slower conduction velocities with all the tissues exhibiting the Wenckebach phenomenon.	Strengths: Robust AV node-specific differentiation protocol that generates physiologically relevant cell populations. Limitations: Still immature phenotypically and protocol generates a mix of core atrioventricular node, atrial transition zone, and lower nodal bundle cells, and so specific investigation into a single cell type would require FACS.

## Data Availability

No new data were created or analyzed in this study.
